# The study of the relationship between food additives and the childhood asthma based on metabolome analysis

**DOI:** 10.3389/fimmu.2025.1671022

**Published:** 2025-09-26

**Authors:** Mingcong Chen, Xinfeng Xu, Xinyao Jiang, Jinyan Hui, Xuna Tao, Yuling Bao, Shuyuan Cao, Qian Wu

**Affiliations:** ^1^ Department of Physical and Chemical Inspection and Nanjing Municipal Key Laboratory for Public Health Laboratory Technology, Nanjing Municipal Center for Disease Control and Prevention of Nanjing Medical University, Nanjing, Jiangsu, China; ^2^ China International Cooperation Center (CCC) for Environment and Human Health and Department of Health Inspection and Quarantine, School of Public Health, Nanjing Medical University, Nanjing, Jiangsu, China; ^3^ Department of Respiratory, Children’s Hospital of Nanjing Medical University, Nanjing, Jiangsu, China

**Keywords:** food additives, childhood asthma, metabolism, helper T cells, dendritic cells, antigen-presenting cells

## Abstract

**Background:**

Epidemiological evidence suggests health risks arise from intake of food additives. This study aims to investigate the mechanisms linking food additives to childhood asthma through a metabolomics strategy.

**Methods:**

A total of 120 children with asthma and 120 control subjects were recruited. Serum concentrations of ten food additives - including cyclamate, neotame, aspartame, sodium saccharin, acesulfame, sucralose, benzoic acid, dehydroacetic acid, sunset yellow, and ponceau 4R - were quantified using UPLC-MS/MS. The associations between food additives and asthma were evaluated by logistic regression and chi-square tests. Serum metabolic profiling was performed by UPLC-MS. Identified asthma-associated metabolites were subsequently analyzed for pathway enrichment and mediation effects. In murine studies, acesulfame, sodium saccharin, sodium benzoate, or their mixtures were co-administered with OVA to C57BL/6 mice. Airway inflammation, IgE, IL-4, IL-17A, immune cell differentiation, and CD4^+^ T cell metabolomics profiles were assessed.

**Results:**

The detection rates for dehydroacetic acid, benzoic acid and sodium cyclamate exceeded 60%. Benzoic acid, dehydroacetic acid and acesulfame were significantly associated with asthma. Mediation analysis identified fourteen metabolites as mediators in the relationship between benzoic acid and dehydroacetic acid, and childhood asthma, including PC(14:0/14:0), LysoPC(17:0), glycerophosphocholine, PC(18:1(9Z)e/2:0), PE(18:2(9Z,12Z)/14:0), glutamic acid, glutamine, GlcCer(d18:1/16:0), sphingosine, sphingosine-1-phosphate, spermine, spermidine, histidine, and acetylcholine. These metabolites were enriched in glycerophospholipid metabolism, β-alanine metabolism, glutathione metabolism, sphingolipid metabolism, arginine and proline metabolism, arginine biosynthesis, and histidine metabolism pathways. In murine models, food additives significantly exacerbated lung tissue inflammation and elevated levels of IgE, IL-4, and IL-17A in both BALF and serum, and also increased eosinophil percentages in BALF. Furthermore, flow cytometry showed significant alterations in Th1/Th2, Th17/Treg and allergic DC/tolerogenic DC balance within the mesenteric lymph node (MLN) and the lung tissue. Metabolomic profiling of CD4^+^ T-cells from the MLN demonstrated that food additives primarily disrupted phenylalanine, tyrosine, and tryptophan biosynthesis, and glycerophospholipid metabolism pathways. This disruption involved key metabolites including PC(36:4), platelet-activating factor, LysoPE(P-16:0), PS(14:0/5-iso PGF2VI), PE(14:1(9Z)/15:0), Na,Na-dimethylhistamine, docosadienoic acid, cyclohexaneundecanoic acid, L-acetylcarnitine, phosphorycholine, Cer(d18:2/20:0), DG(22:1n9/0:0/20:4n6), 5’-methylthioadenosine, L-tyrosine, and N-palmitoyl leucine.

**Conclusion:**

Food additives may aggravate asthma by metabolically dysregulating the homeostasis of helper T-cells and antigen-presenting cells, thereby disrupting immune tolerance.

## 1 Introduction

Food additives are defined as substances that are not normally consumed as food or used as a typical food ingredients, but added to food for a technological purpose such as preserving during manufacturing (1). The Chinese national food standard (GB 2760‐2014) defines 22 categories of food additives, including sweeteners, colorants, preservatives, antioxidants, and emulsifiers. In recent years, the development of the food industry and advances in science have led to a wider use of food additives in ultra-processed foods, while lifestyle changes have driven a sharp increase in their consumption. A study showed that the intake of ultra-processed foods is negatively correlated with age, with children exhibiting higher intake levels (2, 3). Children may be particularly sensitive to the effects of these food additives because their developing metabolic systems may not yet efficiently detoxify them (4, 5). Although commonly used food additives are generally considered safe, they may pose greater health risks to individuals with existing diseases or long-term intake of near-toxic doses of food additives. Several epidemiological studies have demonstrated adverse effects of food additives on children, including asthma/allergy, glucolipid metabolic disorders (6–8), and an increased risk of attention deficit hyperactivity disorder. Therefore, the potential health risks of food additives to children’s health have become an important public health issue.

To date, several epidemiological studies have demonstrated an association between food additives and asthma/allergy. Lemoine et al. conducted a study that confirmed intake of food additives (tartrazine, carmine red, sunset yellow, erythrosine, patent blue V and sodium benzoate) was associated with allergies of the mucous membrane, skin, and intestines in children (9). Lesliam et al. found that methylparaben and propylparaben exposure was associated with an increase in emergency department visits due to asthma attacks during the previous twelve months (10). Ekaterina et al. found that women who drank more artificially-sweetened non-carbonated soft drinks were 1.23 times more likely to have childhood asthma in their offspring compared with women who did not drink (7). However, few studies in China have evaluated the relationship between food additive exposure levels in children and the risk of childhood asthma. Therefore, it’s particularly important to conduct studies on the relationship between food additives and childhood asthma in China.

However, the mechanism of the role of food additives in the development of childhood asthma has not yet been elucidated. A report has indicated that food colorants (tartrazine, sunset yellow, ponceau 4R, and allura red) might promote the oxidation of arachidonic acid and aggravate asthmatic episodes via the upregulation of leukotriene B4 (LTB4) in human neutrophilic granulocytes (11). As an essential tool in systems biology, metabolomics can comprehensively analyze the dynamic relationship between exposure to exogenous substances and endogenous metabolism, providing a new research perspective for revealing the mechanism by which food additives contribute to the development of asthma.

Therefore, the aim of this study was to elucidate the mechanism by which food additives are involved in the development of childhood asthma using metabolomics strategy, to provide a scientific basis for evaluating the risks of food additives and promoting prevention policies of childhood asthma. We first conducted a correlation analysis between food additives and childhood asthma, and then performed a non-targeted metabolic profile of serum samples from children. Multivariate statistical analysis was used to identify asthma-related metabolites, and the potential mediating effects of food additives on asthma pathogenesis were assessed through mediation analysis. Finally, a murine asthma model and an oral tolerance model were established to explore how food additives disturb the differentiation of CD4^+^ T cells and dendritic cells (DCs) through metabolites and ultimately influence the development of asthma.

## 2 Methods and materials

### 2.1 Subjects

The study included 120 asthmatic subjects and 120 control subjects (0- to 15-year-old children), who were recruited from the Children’s Hospital of Nanjing Medical University and the Affiliated Hospital of Nanjing University of Traditional Chinese Medicine from 2020 to 2021, respectively. Control subjects had no history of inflammatory disease, atopy, respiratory-related diseases, or allergies. Asthmatic subjects were diagnosed by physicians during a three-month remission phase and had not taken antibiotics during this period. The Nanjing Medical University Clinical Research Ethics Committee, Nanjing, China, reviewed and approved the protocols of this study (Approval Number: 201902080-1). Written informed consent was obtained from the participants’ parents for the use of samples in this study. Whole blood samples were centrifuged at 5,000 rpm for 10 min. The serum was then collected and stored at -80 °C until analysis.

### 2.2 The analysis of food additives in the serum by UPLC-MS/MS

Sodium saccharin 1,000 mg/mL in water, benzoic acid 1 mg/mL in water, ponceau 4R 0.5 mg/mL in water, and sunset yellow 0.5 mg/mL in water were purchased from National Institute of Metrology, China. Cyclamate 10,000 mg/L in water was purchased from ANPEL Laboratory Technologies (Shanghai) Inc., China, Dehydroacetic acid 1,003 mg/mL in acetonitrile was purchased from ANPEL-TRACE Standard Technical Services (SHANGHAI) Co., Ltd., China. Neotame 1,000 μg/mL in water was purchased from Tan-Mo Quality Inspection Technology Co., Ltd., China. Aspartame 1,000 μg/mL in water was purchased from Beijing Haian Hongmeng Reference Material Technology Co., Ltd., China. Sucralose (purity 99.9%) was purchased from Manhage (Beijing) Biotechnology Co., Ltd., China; acesulfame (purity 99.6%) was purchased from LGC Science (Nanjing) Ltd., China.

First, 150 μL of the serum was mixed with 450 μL of methanol (TEDIA Company, Inc., USA) by using a vortex mixer for 1 minute. The mixture was then centrifuged at 12,000 rpm for 10 minutes. Then the supernatant was concentrated to 100 μL using Refrigerated CentriVap Vacuum Concentrators (Labconco Corporation, USA) for further analysis. An LC-30A high-performance liquid chromatography system (Shimadzu Corporation, Japan) equipped with an Atlantis TM dC18 column (150 mm×2.1 mm, 5 μm, Waters Corporation, USA) and an AB SCIEX QTRAP 4500 mass spectrometer (SCIEX, USA) were used to conduct quantitative analysis of sodium saccharin, cyclamate, acesulfame, sucralose, neotame, aspartame, benzoic acid, dehydroacetic acid, ponceau 4R, and sunset yellow. The column temperature was 40 °C. The injector temperature was set to 4 °C. The injection volume of samples was 5 μL. The mobile phase consisted of 10 mmol/L aqueous ammonium acetate (phase A) and acetonitrile (phase B). The flow rate of mobile phase was 0.3 mL/min. The gradient elution program was provided in **Supplementary Table 1**. In addition, mass spectrometry was performed in electrospray ionization (ESI) heated negative ion mode. The mass spectrometry parameters for food additives were listed in **Supplementary Table 2**. Quantitative analysis was performed using the external standard method, and analyte concentrations were determined based on the established standard curve equation.

### 2.3 The analysis of metabolic profile by UPLC-MS

Fifty microliters of the serum were mixed with 150 μL of methanol and 2 μL of 10 μg/mL tryptophan-D5 internal standard solution (Toronto Research Chemicals, Toronto, Canada) using a vortex mixer for 30 seconds, followed by centrifugation at 4 °C, 12,000 rpm for 15 minutes. The resulting supernatant of 150 μL was mixed with 7.5 μL of 4-Chloro-DL-Phenylalanine internal standard solution (Sigma Aldrich, MO, USA) and held for further analysis. Samples were injected in random order using Xcalibur (version 4.3, Thermo Fisher Scientific Inc., USA). Analysis was performed using UPLC Ultimate 3000 HPLC System (Thermo Fisher Scientific Inc., USA) with Hypersil Gold C18 column (100 mm×2.1 mm, 1.9 μm, Thermo Fisher Scientific Inc., USA) and Orbitrap mass spectrometer (Thermo Fisher Scientific Inc., USA). The column temperature was 40 °C. The injector temperature was 4 °C. The injection volume of samples was 10 μL. The mobile phase was 0.1% formic acid in acetonitrile (phase A) and 0.1% aqueous formic acid (phase B). The flow rate of mobile phase was 0.4 mL/min. The gradient elution program is provided in **Supplementary Table 3**. In addition, the mass spectrometry was conducted using ESI heating and both positive and negative ion modes. The mass spectrometry parameters for untargeted metabolome is provided in **Supplementary Table 4**.

The raw MS data were processed by MS Convert (version 3.0, ProteoWizard, CA, USA) and XCMS Online (https://xcmsonline.scripps.edu). The data were then normalized to the total peak area for each sample, and peaks that did not meet the quality control criteria were excluded. Candidate peaks were selected based on *q* values (calculated using R, version 4.2.2, The R Foundation, Vienna, Austria) and variable importance in projection (VIP) values (computed using SIMCA, version 14.1, Umetrics, Umea, Sweden). These candidate peaks were subsequently matched against the Human Metabolome Database (HMDB, https://hmdb.ca) for metabolite identification. Finally, asthma-related metabolic pathways were annotated using MetabAnalyst 6.0 (https://www.metaboanalyst.ca).

### 2.4 Animals

All animal experiments were approved by the Nanjing Medical University Institutional Animal Care and Use Committee (Approval Number: IACUC-2410056). Female C57BL/6 mice (approximately 6 weeks old, weighing 20 g, Animal Core Facility of Nanjing Medical University, Jiangsu, China) were used in this study and housed in an animal facility (23 ± 1 °C, 50 ± 10% RH) (12). According to the developmental correlation described by Dutta et al., 6-week-old (42-day-old) C57BL/6 mice are equivalent to a human in late adolescence (13). Seventy mice were randomly assigned to seven groups (n=10). Mice in the control group were treated with 1 × PBS. To establish the 23-day acute asthma model, mice were sensitized by intraperitoneal injection once weekly for three weeks with 2.5 mg/kg of OVA (Merck KGaA, Germany) and 15 mg/kg of aluminum hydroxide (Al(OH)_3_) adjuvant (Macklin Inc., Shanghai, China). One week after the last sensitization (14), mice were challenged with 5 mg/kg of OVA via intranasal delivery for 3 consecutive days. For the oral tolerance model, mice were gavaged with 50 mg/kg of OVA for seven consecutive days, one week prior to the first sensitization. Subsequent procedures were the same as those in the asthmatic group. Food additives were administered at induction of oral tolerance. Mice were gavaged with 1 g/kg of acesulfame, 600 mg/kg of sodium saccharin, 600 mg/kg of sodium benzoate and a mixture of these food additives. The Meeh-Rubner formula was used to convert acceptable daily intake (ADI) for humans to ADI for mice and a dose ten times this value was applied (15). Additionally, acesulfame (97% purity), sodium saccharin (98% purity) and sodium benzoate (99.5% purity) were purchased from Macklin Inc., Shanghai, China.

### 2.5 Total IgE, IL-4 and IL-17A analysis

The total concentration of IgE, IL-4 and IL-17A in the BALF and serum of mice was analyzed using Mouse IgE Precoated ELISA Kit, Mouse IL-4 Precoated ELISA Kit and Mouse IL-17A Precoated ELISA Kit (DAKEWE, Shenzhen, China). Absorbance was measured at 450 nm (measurement) and 620 nm (reference) using an Infinite M2000 plate reader.

### 2.6 Neutrophilic granulocytes and eosinophil granulocytes counting

The proportions of neutrophilic granulocytes and eosinophil granulocytes were measured using an ADVIA 2120i System (Siemens, Germany) in the BALF.

### 2.7 Hematoxylin and eosin staining

The lung tissue was fixed in the 4% paraformaldehyde solution (Jiangsu Keygen Biotech Co., Ltd., Jiangsu, China). Subsequently, the tissue was embedded in paraffin and cut into sections 3-5 μm thick. The sections were then stained with H&E and dehydrated and mounted. Sections were scanned using PANNORAMIC SCAN Digital Scanner (3DHISTECH Ltd., Hungary). Five non-overlapping 500 μm × 500 μm regions of interest were randomly selected in each group and exported as digital images. Nuclei of inflammatory cells within each image were identified using the StarDist Python library (version 0.9.1) (16–18), in conjunction with a pre-trained model optimized for nuclear segmentation in 2D H&E-stained pathological sections (19, 20). The number of inflammatory cells per image was subsequently quantified and statistically summarized.

### 2.8 Flow cytometer analysis

The fluorescent-dye conjugated antibodies used in this study were as follows: BUV737-conjugated anti-IFN-γ, BV421-conjugated anti-IL-4, BV786-conjugated anti-CD11c, BV510-conjugated anti-CD103, PE-Cy7-conjugated anti-CD45, BV605-conjugated anti-CD25, and Fixable Viability Stain 780 (all from Becton, Dickinson and Company, USA); PerCP-Cy5.5-conjugated anti-CD4, PE-conjugated anti-Foxp3, and AF488-conjugated anti-IL-17A (all from BioLegend, Inc., USA); and BV395-conjugated anti-CD11b (from Thermo Fisher Scientific Inc., USA).

To obtain helper T cells, the single-cell suspension derived from MLN and lung tissue of mice was mixed with a Cell Activation Cocktail (BioLegend, Inc., USA) and cultured at 37 °C with 5% CO2 for 6 hours. Before staining, the samples were mixed with Fc block antibody (Becton, Dickinson and Company, USA) and kept at 4 °C for 10 minutes to prevent the non-specific binding of antibody. The samples were then stained with anti-CD45, anti-CD25 and anti-CD4 at 4 °C in the dark for 30 minutes. Fixable Viability Stain 780 was used to eliminate dead cells. After that, Foxp3/Transcription Factor Staining Buffer Set (Thermo Fisher Scientific Inc., USA) was used to conduct fixation and permeabilization treatment. Finally, the samples were stained with anti-IFN-γ, anti-IL-4, anti-IL-17A and anti-Foxp3 at 4 °C in the dark for 30 minutes. To obtain DCs, the samples were mixed with Fc Block antibody and kept in 4 °C for 10 minutes. Afterward, the samples were stained with anti-CD11b, anti-CD11c, anti-CD103, anti-CD45, and anti-CX3CR1 at 4 °C in the dark for 30 minutes.

Finally, the sample was detected by the FACSymphony A5 SORP Cell Analyzer (Becton, Dickinson and Company, USA) and the data were analyzed with FlowJo (version 10.9.0, Becton, Dickinson and Company, USA).

### 2.9 The analysis of metabolic profile in CD4^+^ T cells by UPLC-MS

The single-cell suspension derived from MLN was processed using Mouse CD4^+^ Cell Separation Kit (RWD Life Science Co., Ltd., Shenzhen, China) to enrich CD4^+^ T cells. The enriched cells were then homogenized with zirconium beads (Wuhan Servicebio Technology Co., Ltd., Wuhan, China) and 100 μL of 80% methanol using a grinder for 5 minutes, followed by centrifugation at 4 °C, 12,000 rpm for 15 minutes for metabolome analysis.

### 2.10 Statistical analysis

Data were presented as mean ± SD. Differences in the levels of food additives in the serum of children, the expression of inflammatory factors in the BALF of mice, the proportion of neutrophilic granulocytes and eosinophil granulocytes in the BALF of mice, and the proportion of helper T cells and DCs in the tissues of MLN and lung of mice among each group were analyzed using Welch t test in GraphPad Prism (version 10.4.1, GraphPad Software). Differences in age and gender among participants were analyzed using chi-square test and independent sample t test, respectively by SPSS Statistics (version 26, IBM, USA). For food additives with detection rates exceeding 50%, their association with childhood asthma was assessed using logistic regression. For food additives with detection rates ≤ 50%, chi-square tests were employed. In addition, mediation analysis was conducted by fitting two separate models: a model for childhood asthma (outcome model, Equation 1) and a model for metabolites (mediator model, Equation 2).


(1) $$\begin{array}{c} \log\left(\pi i\right)=\theta 0+\theta 1\left(Food\;additives\right)i+\theta 2\ln\left(Metabolites\right)i+\theta 3Ci \end{array}$$

(2) $$\begin{array}{c} E\left(\ln\left(Metabolites\right)i\right)=\beta 0+\beta 1\left(Food\;additives\right)i+\beta 2Ci \end{array}$$

In mediation analysis, *πi* represents the probability of asthma occurrence for subject *i*, with *Ci* corresponding to their covariate matrix. The asthma model and the mediator model were combined to estimate the direct, indirect, and total effects for the mediation analysis. Asthma status was modeled using multivariable logistic regression with robust variance estimators. Adjusted regression coefficients were estimated (Equation 1). Mediator variables were analyzed using multivariable linear regression (Equation 2). All mediation analyses were implemented using the R mediation package, and statistical significance was defined as two-tailed P< 0.05.

## 3 Results

### 3.1 The levels of food additives in the serum were associated with childhood asthma

**Figure 1:**
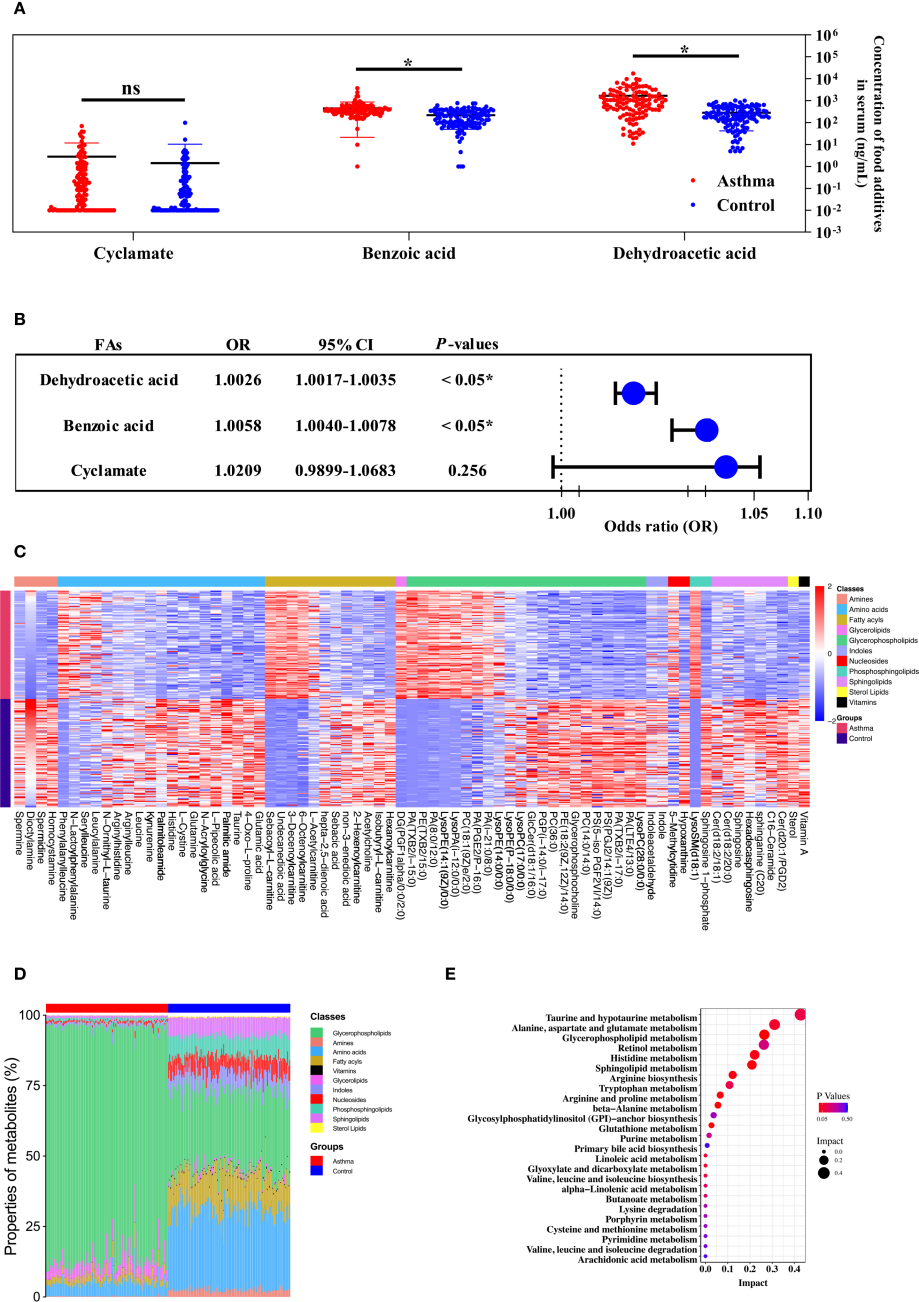
The metabolic profile of the serum was contributed to the relationship between the level of food additives and childhood asthma. **(A)** The concentration and distribution of food additives in the serum of children with the asthmatic group and the control group. **(B)** Forest plot of the correlation between food additive concentration in the serum of children and childhood asthma. **(C)** Heatmap visualization of differentially metabolites between the asthmatic group and the control group. **(D)** The comparison of major metabolite categories represented as percentage of total detected metabolites. **(E)** Pathway enrichment analysis of differential metabolites mapped to KEGG metabolic pathways.

In this study, the characteristics of the children were summarized in **Supplementary Table 5**. The mean age was 4.53 ± 2.49 years in the asthmatic group and 5.00 ± 2.96 years in the control group. There were no significant differences in age and gender between the asthmatic and control group. **Table 1** lists food additives detected in this analysis, the standard curve equations, the *R* ^2^ values, LOD, spiked recovery rates, detection rates, and concentration ranges of food additives. Dehydroacetic acid was detected at the highest rate (99.58%), followed by benzoic acid (99.17%) and cyclamate (69.17%). Acesulfame, sodium saccharin, sucralose, sunset yellow, and ponceau 4R were detected at low frequencies, while aspartame and neotame were not detected. **Figure 1A** shows that the average concentrations of benzoic acid and dehydroacetic acid were significantly higher in the asthmatic group than in the control group. Significant associations were found between childhood asthma and exposure to dehydroacetic acid, benzoic acid, and acesulfame, as determined by logistic regression and chi-square tests, respectively (**Figure 1B**, **Table 2**).

**Table 1:** Linear equations, LOD and spike recoveries of food additives.

Categories	Food additives	Linear equations	r^2^	LOD (ng/mL)	Spike recoveries (%, Min-Max)	Total detection rate (%)	Detection rate in asthmatic group (%)	Detection rate in control group (%)	Total range of concentration (ng/mL)
Sweeteners	Cyclamate	y = 5.64621e4x - 3.03002e4	0.99901	0.01	60.07-99.89	69.17	82.5	55	ND-84.956
	Neotame	y = 15905.45243x - 105.76862	0.99911	0.02	61.05-103.26	0	0	0	ND
	Aspartame	y = 2506.70153x + 132.47755	0.99961	0.25	80.54-85.60	0	0	0	ND
	Sodium Saccharin	y = 1900.31933x - 489.66636	0.99945	0.5	86.83-88.52	10.42	15.83	5	ND-61.674
	Acesulfame	y = 199.00604x - 15.02552	0.99705	1.5	90.82-99.32	35.83	54.17	17.5	ND-5723.375
	Sucralose	y = 123.97516 x + 135.35927	0.9992	10	89.51-96.29	3.75	5.83	1.667	ND-72.942
Preservatives	Benzoic acid	y = 3961.81557x - 11634.74861	0.9988	1	105.62-121.40	99.17	100	98.33	ND-2642.595
	Dehydroacetic acid	y = 1999.87275x + 18.18439	0.99974	5	74.19-88.41	99.58	100	99.17	ND-17475.311
Colorants	Sunset yellow	y = 7.77638e4x + 10488.10583	0.9979	0.01	50.62-59.12	1.667	2.5	0.833	ND-0.924
	Ponceau 4R	y = 2671.12322x + 1176.24352	0.99754	0.25	63.22-77.37	1.25	0	2.5	ND-5.485

ND, Not Detected.

**Table 2:** Chi-square test on the association between food additives and childhood asthma.

Food additives	Groups	(+)	(-)	Detection rates (%)	χ^2^	*P*	OR	95% CI
Acesulfame	Asthma	59	61	49.17	35.859	< 0.001	2.128	1.688-2.682
	Control	16	104	13.33				
	Total	75	165	31.25				
Sodium saccharin	Asthma	19	101	15.83	3.177	0.075	1.369	1.015-1.846
	Control	10	110	8.33				
	Total	29	211	12.08				
Sucralose	Asthma	5	115	4.17	1.538	0.215	1.696	1.159-2.482
	Control	1	119	0.83				
	Total	6	234	2.5				
Sunset yellow	Asthma	3	117	2.5	0	1.000	1.205	0.582-2.494
	Control	2	118	1.67				
	Total	5	235	2.08				

### 3.2 The mediating effects of metabolites in the relationship between food additives and childhood asthma

Distinct patterns in the concentration and composition of differential metabolites were observed between the asthmatic and control groups (**Figures 1C, D**). Seventy-three statistically significant asthma-associated metabolites were identified using multivariate statistical analysis and HMDB matching (**Supplementary Table 6**). Enriched pathways identified from the differential metabolites are shown in **Figure 1E**. Mediation analysis was performed to evaluate whether metabolites from the enriched pathways mediate the association between food additives and childhood asthma (**Supplementary Table 7**). As a result, the metabolites that mediated the association between benzoic acid and dehydroacetic acid exposure and childhood asthma included PC(14:0/14:0), LysoPC(17:0), glycerophosphocholine, PC(18:1(9Z)/2:0), PE(18:2(9Z,12Z)/14:0), glutamic acid, glutamine, histidine, spermine, spermidine, GlcCer(d18:1/16:0), sphingosine, sphingosine-1-phosphate, and acetylcholine. This suggested that these metabolites may be particularly affected by benzoic acid and dehydroacetic acid, potentially disrupting the differential metabolic pathways associated with childhood asthma.

### 3.3 Food additives exacerbated inflammation in murine asthma model

**Figure 2:**
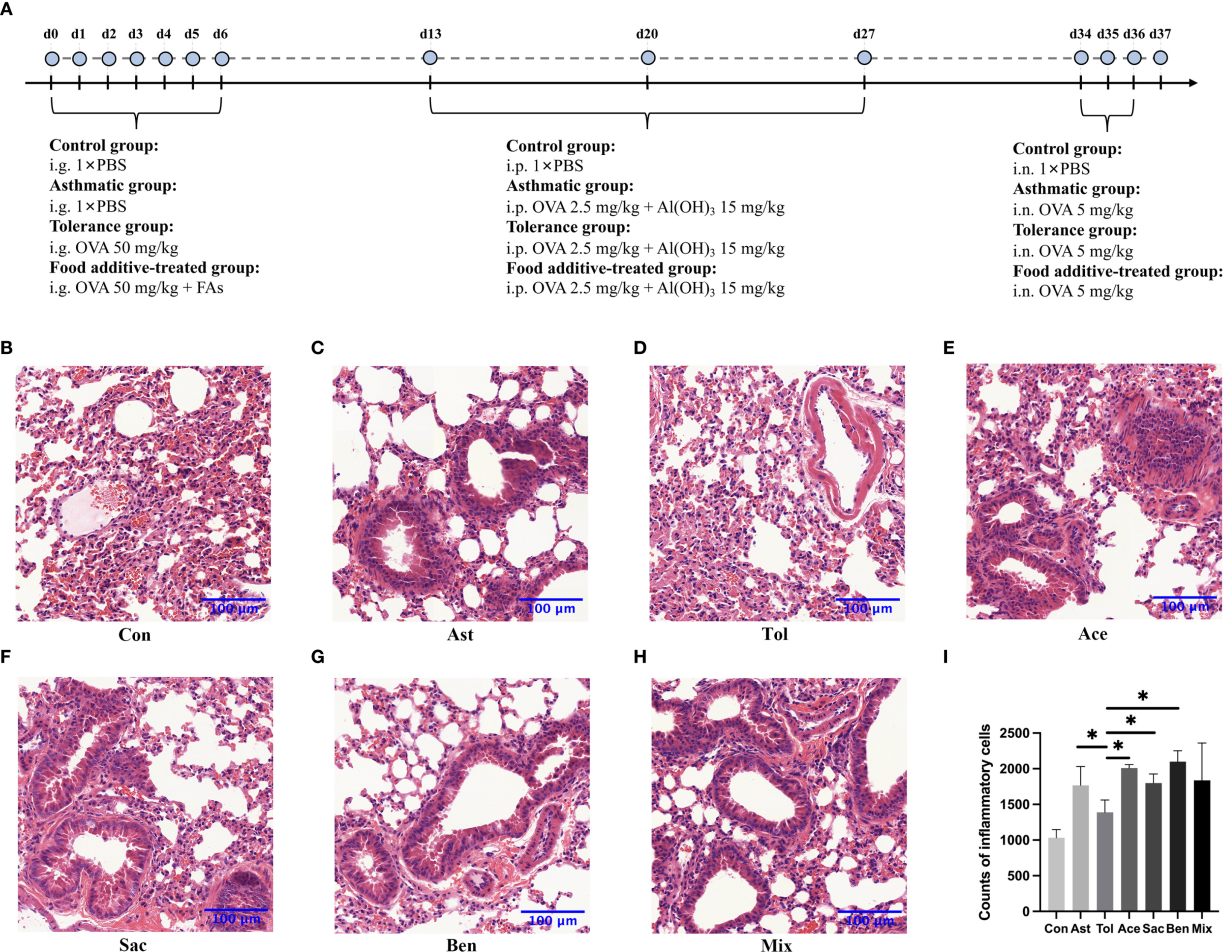
The flowchart of animal experimentation and the morphology of the lung tissues by H&E staining. The flowchart of animal experimentation was present in **(A)**. And the results of morphology of the lung tissues by H&E staining were as follows: control group **(B)**, asthmatic group **(C)**, tolerance group **(D)**, acesulfame-treated group **(E)**, sodium saccharin-treated group **(F)**, sodium benzoate-treated group **(G)** and mixture-treated group **(H)**. The counts of inflammatory cells in each group by H&E staining **(I)**, Con, the controls. Ast, the asthma model. Tol, the oral tolerance in asthma model. Ace, acesulfame-treated group. Sac, sodium saccharin-treated group. Ben, sodium benzoate-treated group. Mix, mixture-treated group (Ace + Sac + Ben). n=4. *P < 0.05.

**Figure 3:**
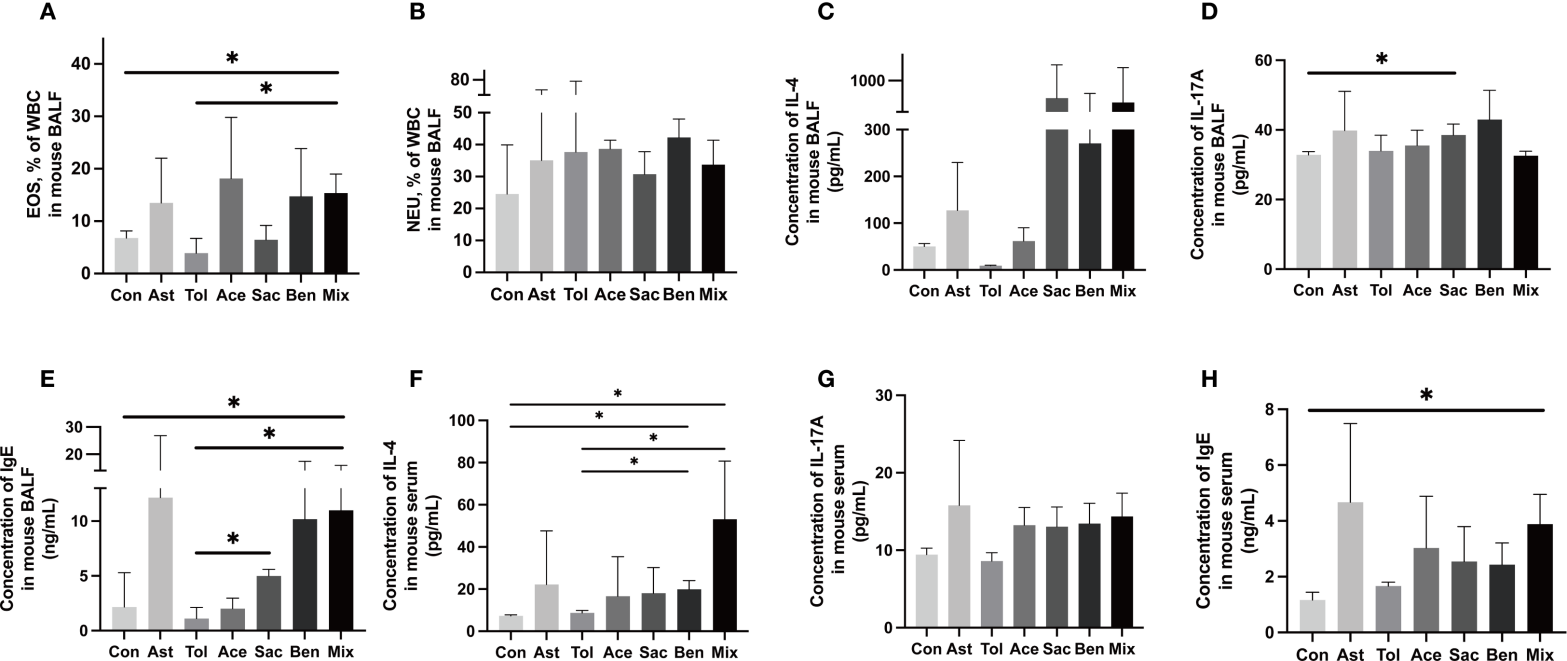
Inflammatory characterization in the BALF and serum.The proportion of eosinophil granulocyte (EOS) **(A)**, neutrophil granulocytes (NEU) **(B)** and the concentration of IL-4 **(C)**, IL-17A **(D)** and IgE **(E)** in the BALF. The concentration of IL-4 **(F)**, IL-17A **(G)** and IgE **(H)** in the serum. Con: the controls. Ast: the asthma model. Tol, the oral tolerance in asthma model. Ace, acesulfame-treated group. Sac, sodium saccharin-treated group. Ben, sodium benzoate-treated group. Mix, mixture-treated group (Ace + Sac + Ben). Data was represented as mean ± SD (n=4), *P < 0.05.

The murine experimental design was shown in **Figure 2A**. Histopathological analysis showed that bronchial constriction and peribronchovascular inflammatory cell infiltration were observed in asthmatic and food additive-treated groups (**Figures 2B-H**). Quantitative analysis of inflammatory cells in tissue sections for each group is presented in **Figure 2I** and **Supplementary Table 8**. Compared with both the control and oral tolerance groups, food additive-treated groups exhibited a significant increase in inflammatory cells in the sampled tissue areas. The proportion of eosinophil granulocytes in the BALF, as well as the levels of IL-17A in the BALF, IL-4 in the serum, and IgE in both the BALF and the serum, were significantly elevated in the food additive-treated groups compared with the control group (**Figure 3**).

### 3.4 Food additives disrupted immune cell differentiation pathways in murine models

**Figure 4:**
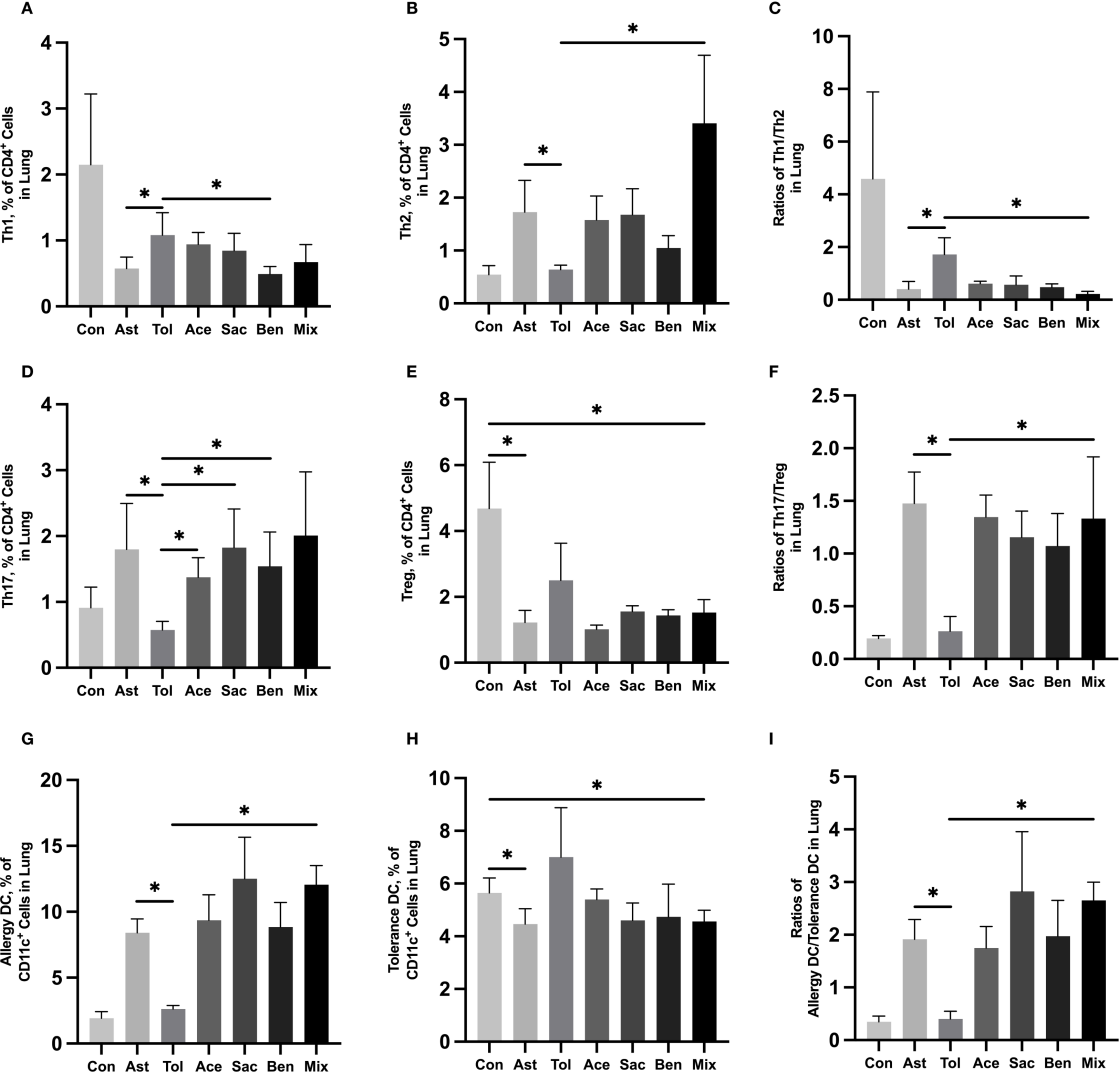
Immune cell profiling in murine lung tissue analyzed by flow cytometry Murine lung tissue was used to perform the flow cytometry. Th1 cell proportion **(A)** and Th2 cell proportion **(B)** in total T cells, and Th1/Th2 ratio **(C)**. Th17 cell proportion **(D)** and Treg cell proportion **(E)** in total T cells, and Th17/Treg ratio **(F)**. Tolerance DC proportion **(G)** and Allergy DC proportion **(H)** in total DCs, and Allergy DC/tolerance DC ratio **(I)**. Con, the controls. Ast, the asthma model. Tol, the oral tolerance in asthma model. Ace, acesulfame-treated group. Sac, sodium saccharin-treated group. Ben, sodium benzoate-treated group. Mix, mixture-treated group (Ace + Sac + Ben). Data was represented as mean ± SD (n=4), *P < 0.05.

**Figure 5:**
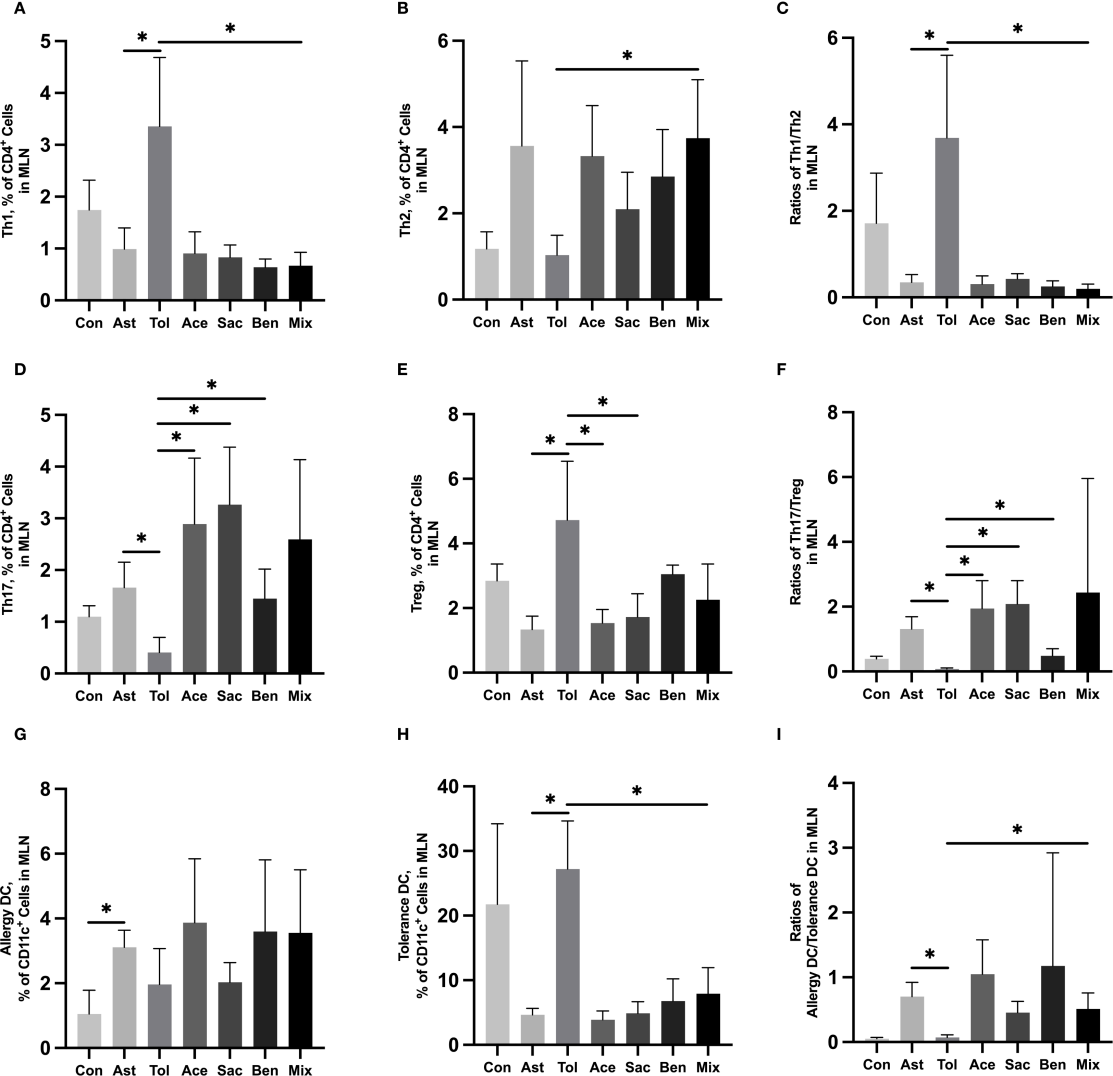
Immune cell profiling in murine MLN tissue analyzed by flow cytometryMurine MLN tissue was used to perform the flow cytometry. Th1 cell proportion **(A)** and Th2 cell proportion **(B)** in total T cells, and Th1/Th2 ratio **(C)**. Th17 cell proportion **(D)** and Treg cell proportion **(E)** in total T cells, and Th17/Treg ratio **(F)**. Tolerance DC proportion **(G)** and Allergy DC proportion **(H)** in total DCs, and Allergy DC/tolerance DC ratio **(I)**. Con, the controls. Ast, the asthma model. Tol, the oral tolerance in asthma model. Ace, acesulfame-treated group. Sac, sodium saccharin-treated group. Ben, sodium benzoate-treated group. Mix, mixture-treated group (Ace + Sac + Ben). Data was represented as mean ± SD (n=4), *P < 0.05.

The gating strategies for helper T cells (Th1: CD4^+^IFN-γ^+^; Th2: CD4^+^IL-4^+^; Th17: CD4^+^IL-17A^+^; Treg: CD4^+^CD25^+^FOXP3^+^) and DCs (allergic DC: CD11c^+^CD11b^+^CD103^+/-^; tolerogenic DC: CD11c^+^CD11b^-^CD103^+^) were shown in **Supplementary Figure 1**. In lung tissue, the proportions of Th2 cells, Th17 cells, and allergic DCs were significantly increased in the food additive-treated groups compared with the oral tolerance group. The Th1/Th2 ratio was significantly lower, while the Th17/Treg ratio and the allergic DC/tolerogenic DC ratio were significantly higher in the food additive-treated groups than in the oral tolerance group (**Figures 4A-I**, **Supplementary Table 9**). Similar changes in Th cell and DC subset proportions were observed in MLN tissue (**Figures 5A-I**, **Supplementary Table 10**).

### 3.5 Food additives disrupted metabolism of MLN CD4^+^ T cells in murine models

**Figure 6:**
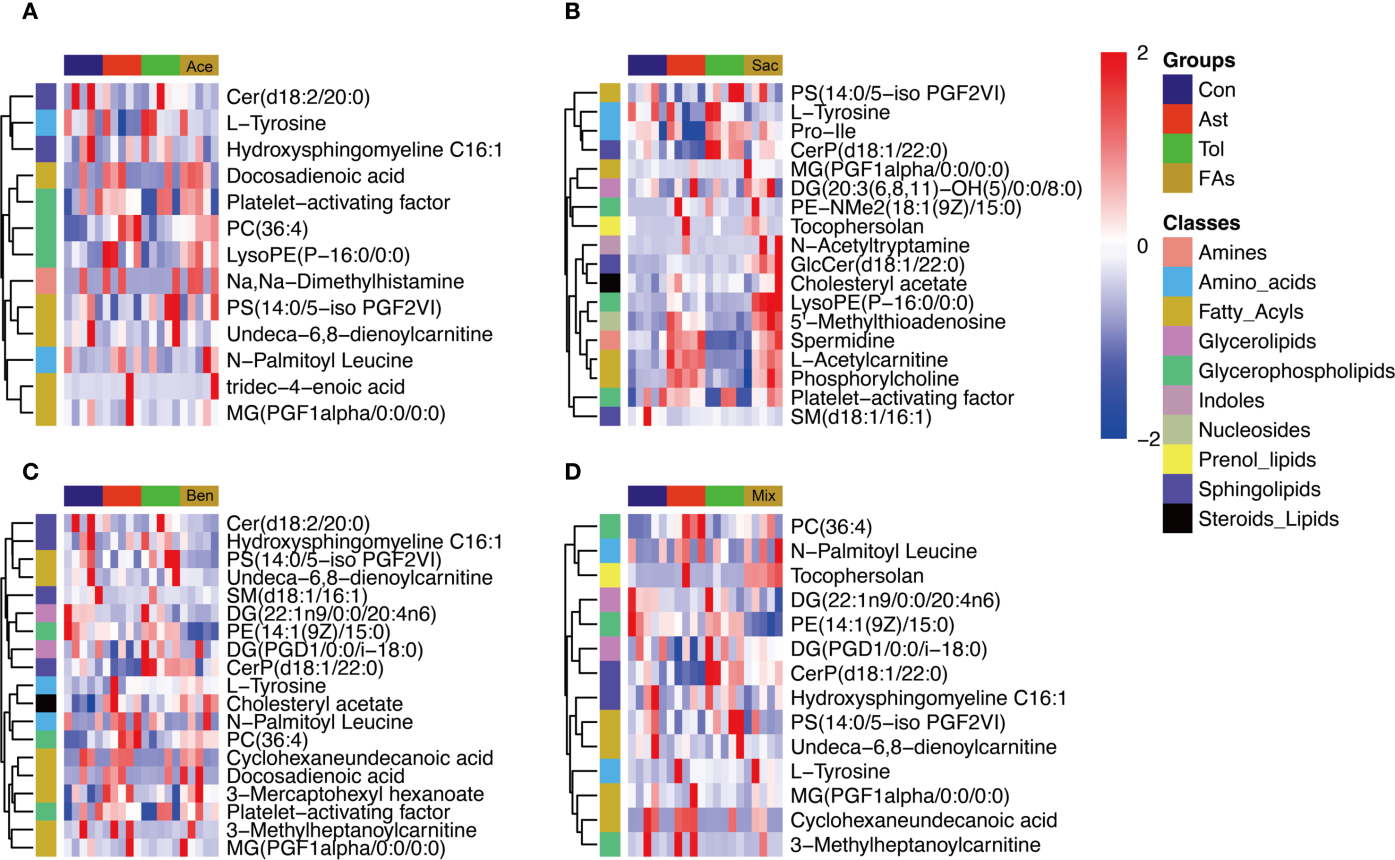
Heatmap visualization of differentially metabolites of murine CD4^+^ cell affected by food additives. The differential metabolites affected by acesulfame **(A)**, sodium saccharin **(B)**, sodium benzoate **(C)** and mixture **(D)**. Con, the controls. Ast, the asthma model. Tol, the oral tolerance in asthma model. Ace, acesulfame-treated group. Sac, sodium saccharin-treated group. Ben, sodium benzoate-treated group. Mix, mixture-treated group (Ace + Sac + Ben). n=5.

**Figure 7:**
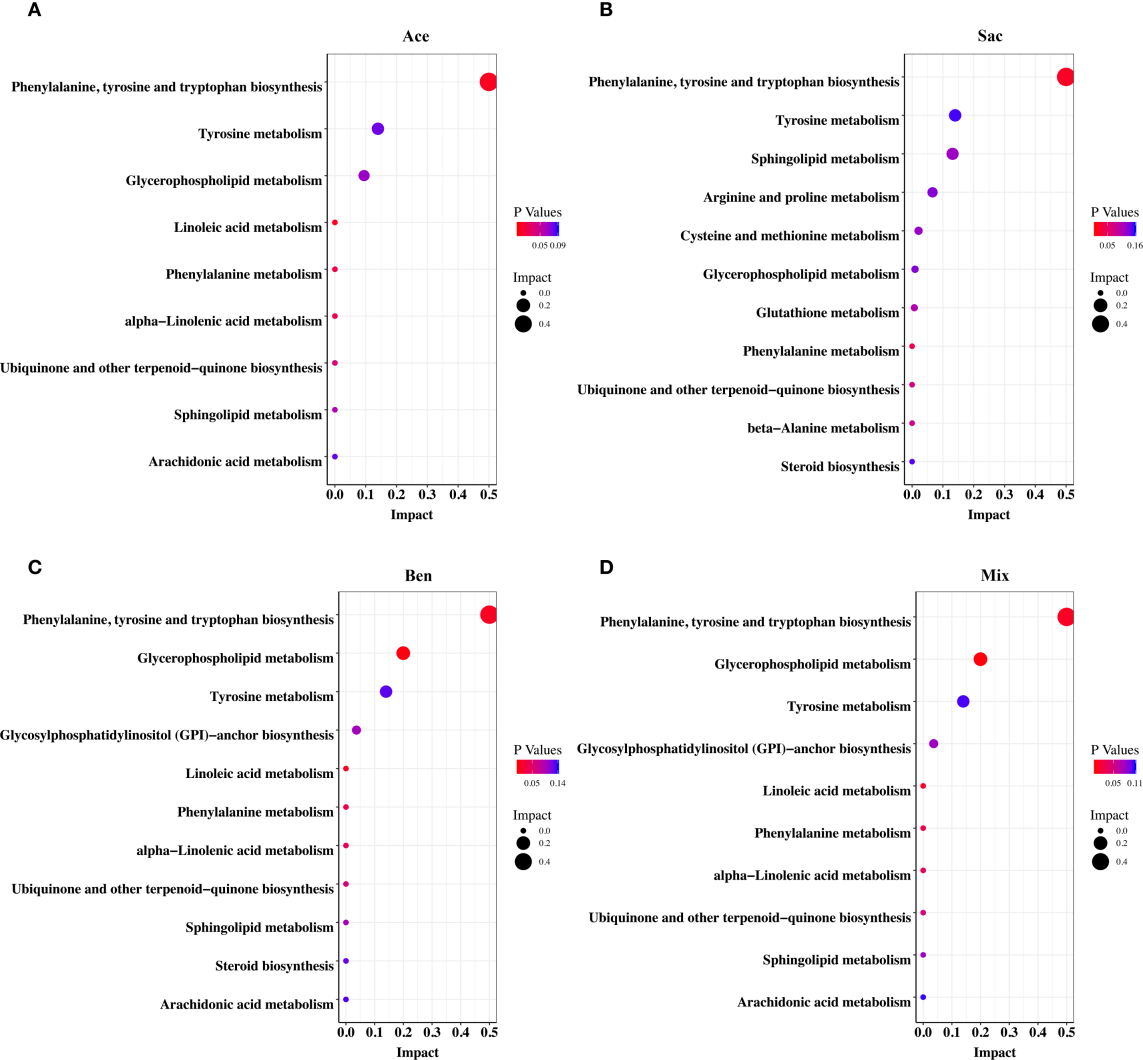
Pathway enrichment analysis of differential metabolites of murine CD4^+^ cell in MLN affected by food additives. The differential metabolites were mapped to KEGG metabolic pathways and the differential metabolites affected by acesulfame **(A)**, sodium saccharin **(B)**, sodium benzoate **(C)** and mixture **(D)**. Con, the controls. Ast, the asthma model. Tol, the oral tolerance in asthma model. Ace, acesulfame-treated group. Sac, sodium saccharin-treated group. Ben, sodium benzoate-treated group. Mix, mixture-treated group (Ace + Sac + Ben). n=5.

The above results indicated that food additives can induce abnormal differentiation of helper T cells, thereby contributing to asthma development. Therefore, CD4^+^ T cells were isolated from the MLN and subjected to non-targeted metabolomics analysis. Differential metabolites were identified using fold change thresholds (<0.8 or >1.2), as presented in **Supplementary Table 11**. **Figure 6** shows heatmaps of the differential metabolites among groups. Compared to both the control and oral tolerance groups, in the acesulfame-treated group, the levels of glycerophospholipids, amines, and fatty acyls increased, while Cer(d18:2/20:0) decreased (**Figure 6A**). In the sodium saccharin-treated group, the levels of glycerophospholipids, nucleotides, amines, and specific lipid metabolites increased, while L-tyrosine decreased (**Figure 6B**). In the sodium benzoate-treated group, the levels of the glycerophospholipids, and the fatty acyls increased, while Cer(d18:2/20:0), PS(14:0/5-iso PGF2VI), PE(14:1(9Z)/15:0), and DG(22:1n9/0:0/20:4n6) decreased (**Figure 6C**). In the mixed additive-treated group, the levels of PC(36:4), N-palmitoyl leucine, and cyclohexaneundecanoic acid increased, while PS(14:0/5-iPF2α-VI), PE(14:1(9Z)/15:0), and DG(22:1n9/0:0/20:4n6) decreased (**Figure 6D**). Pathway enrichment analysis is shown in **Figures 7A-D**. Significant alterations in glycerophospholipid metabolism, phenylalanine, tyrosine, and tryptophan biosynthesis pathways were observed among food additives-treated groups.

## 4 Discussion

The frequency of dietary exposure to food additives in children is increasing, while the prevalence of childhood asthma continues to rise. This underscores the critical need to clarify how these food additives contribute to asthma development. Our data suggest that metabolic disruptions caused by food additives disturbed immune cell differentiation, ultimately exacerbating asthma symptoms. Collectively, these findings elucidate a potential mechanism by which food additive exposure may contribute to the pathogenesis of childhood asthma.

A study in China had investigated the levels and distribution of sweeteners and preservatives in the serum of children. For example, You et al. found that serum levels of acesulfame and cyclamate among children under 10 years old (n=5696 from 15 provinces across China) were higher than those in other age groups (21). In our study, the average serum levels of benzoic acid and dehydroacetic acid were significantly higher in the asthmatic children than in the control subjects. Precise quantification of food additive levels in serum is essential for assessing human exposure. Food additives exhibit distinct structural characteristics that influence their analytical detection. Sulfonamide-based sweeteners (e.g., sodium saccharin, cyclamate and acesulfame) are predominantly analyzed by UPLC-MS/MS, which accounts for 38% of sulfonamide detection methods according to Scopus (22, 23). In contrast, sucralose requires measurement via differential refractive index detection (RID) or UV-Vis spectrophotometry after derivatization due to the lack of significant ultraviolet (UV) absorption (24, 25). Colorants such as tartrazine, ponceau 4R, sunset yellow, and allura red contain strong chromophores (e.g., azo groups, phenyl rings, sulfonic acid groups), making them amenable to liquid chromatography with ultraviolet detection (LC-UV) as prescribed by Chinese national standards (GB 5009.35-2023). To address the need for high-throughput food additive screening while accommodating limited sample volumes, an ultra-high performance liquid chromatography-tandem mass spectrometry (UPLC-MS/MS) method was developed in this study. This approach enables rapid, simultaneous quantification of the aforementioned food additives with high accuracy. This high-throughput method is expected to improve the assessment of food additive exposure levels in children. Serum concentrations can reflect systemic and target organ exposure levels. Therefore, the serum concentrations of various food additives were measured in this study to assess the potential effects. Additionally, the exposure concentrations of food additives within the body are influenced by their intake and metabolism. Sweeteners, colorants, and preservatives are among the most frequently consumed food additives. A Brazilian market survey of 9856 food products found preservatives and colorants to be the most prevalent categories, accounting for 28.9% and 27.8% of food additives, respectively, while sweeteners constituted 10.8% (26). Preliminary investigations in this study examined food additive labeling on products sold near primary schools in six districts of Nanjing (Qinhuai, Yuhuatai, Jianye, Gulou, Xuanwu, and Qixia District). Products included including instant noodles, puffing foods, candies, beverages, bakery products, sausages, stewed meats, preserved fruits, seasoned flour products and bean products. This investigation identified colorants, sweeteners, and preservatives as the top categories, representing 35.3%, 22.8% and 19.0% of food additives, respectively (data not shown). Some readily metabolized or poorly absorbed food additives typically exhibit low detection rates in serum samples. For example, peptide sweeteners such as aspartame and neotame are rapidly hydrolyzed in the human intestine and are not absorbed intact into the bloodstream (27). Hence, neither aspartame nor neotame were detected in serum samples in this study. Furthermore, food additives like sucralose, sunset yellow, and ponceau 4R, possessing molecular weights exceeding 200 Da, exhibit limited absorption across the intestinal mucosa (28). Their inherent chemical stability also impedes degradation into smaller, absorbable molecules (29, 30). Hence, a significant proportion of these compounds is excreted unchanged in feces, resulting in low serum detection rates.

Notably, case reports confirmed that sodium benzoate triggers allergic reactions such as contact urticaria and exacerbates asthma symptoms in children (31, 32). Yamashita et al. found through *in vivo* experiments that acesulfame and sodium saccharin altered the differentiation and migration of DCs, prevented the acquisition of oral tolerance, and enhanced the food allergy development (12). DCs, antigen-presenting cells (APCs), play a central role in inducing helper T cell differentiation and initiating and sustaining Th2 immune responses in the airways (33). Furthermore, CD4^+^ helper T-cell subsets (Th1, Th2, and Th17) are critically involved in the pathogenesis of allergic and asthmatic responses through the secretion of inflammatory cytokines, whereas the diminished Treg populations impair immune homeostasis (34). Elisabeth et al. observed in human peripheral blood mononuclear cell cultures that sodium benzoate suppressed Th1-type immunity by reducing neopterin levels and the kynurenine/tryptophan ratio (35), which may potentiate excessive Th2 responses and disrupt immune homeostasis. Additionally, ponceau 4R can act as an antigen, directly triggering immune activation and lymphocyte differentiation (36).Therefore, we postulated that the intake of food additives may disturb the differentiation and migration of DCs, further inhibiting the function of regulatory T cells, thereby preventing the acquisition of oral tolerance and ultimately leading to the development of food allergy symptoms. Our study demonstrated that acesulfame, sodium saccharin, and sodium benzoate can perturb T cell differentiation, decreasing the Th1/Th2 ratio while increasing the Th17/Treg ratio and the allergic DC/tolerogenic DC ratio.

Evidence suggested that the colorants (sunset yellow, ponceau 4R, allura red, and tartrazine) stimulated the synthesis of the metabolites LTB4 and F2-isoprostanes by neutrophilic granulocytes in the blood. LTB4 is implicated in asthma induction, and F2-isoprostanes are associated with oxidative stress (11). Consequently, we proposed that food additives might disturb metabolite levels, and these metabolites might mediate the process by which food additives induce asthma. Mediation analysis in this study identified significant mediation effects for specific metabolites in the pathway linking benzoic acid, dehydroacetic acid, or both to asthma. For example, phosphatidylcholine (PC), a key component of both cellular and mitochondrial membranes, plays a crucial role in maintaining membrane integrity (37). An animal study demonstrated elevated PC levels in the lungs of asthmatic subjects compared to controls. In contrast, PC levels are reduced in the mitochondrial membranes of airway epithelial cells in asthma. This reduction leads to mitochondrial dysfunction and enhanced apoptosis of these cells (38). LysoPC, generated by phospholipase A2 through the hydrolysis of PC (39), is a key mediator in inflammation. It mediates the chemotaxis of proinflammatory macrophages and sustains the proinflammatory M1 macrophage phenotype (40). Similar to the deficiency of PC, insufficient phosphatidylethanolamine (PE) can also induce mitochondrial dysfunction in airway epithelial cells, contributing to asthma pathogenesis (41). Histamine, an inflammatory mediator derived from the decarboxylation of histidine, directly mediates asthma symptoms such as bronchial smooth muscle contraction and airway constriction (42). Histamine has been implicated in DNA methylation processes during asthma development (43). Sphingosine-1-phosphate (S1P) activates sphingosine-1-phosphate receptor 2 to stimulate the degranulation of mast cell, resulting in the release of inflammatory mediators including prostaglandins, leukotrienes, and platelet-activating factor (44). Elevated S1P levels also promote Th2 responses, increasing Th2, Th17 and macrophage populations. Acetylcholine stimulates cholinergic muscarinic 3 receptor, inducing bronchial smooth muscle contraction and consequent asthma symptoms (45). To further investigate the impact of food additives on immune-related metabolism, we conducted a non-targeted metabolomics analysis of CD4^+^ T cells isolated from the MLN tissue of mice. The results demonstrated that food additives significantly altered the levels of multiple metabolites within CD4^+^ T cells. This mechanism potentially contributing to the observed reduction in L-tyrosine levels (46, 47). Collectively, these findings suggest that alterations in glycerophospholipid, fatty acyl, and amino acid metabolite levels may promote Th cell polarization toward inflammatory subsets such as Th2 cells. This shift could subsequently drive dysregulation of various inflammatory factors within corresponding Th cell populations.

Food additives first interact with the intestinal immune environment upon oral ingestion. Subsequently, they could potentially influence the immune milieu of distal organs and tissues by the gut-lung axis, including the respiratory system. Evidence indicates that immune cells, inflammatory factors, metabolites, and even enteric bacteria may translocate from the intestine to the respiratory system via this axis. Increased intestinal permeability is a primary mechanism facilitating bacterial translocation. For instance, Aparna et al. demonstrated using Caco-2 cell permeability assays that low concentrations (100 µM) of sucralose and aspartame activate Taste 1 Receptor Member 3 receptors, thereby increasing intestinal permeability (48). This compromised barrier function may permit enteric bacteria to translocate to the respiratory system, potentially activating local immune responses (49). An animal study showed that intestinal Th17 cells can undergo long-distance migration to the lungs via the gut-lung axis, contributing to pulmonary inflammation (50). The intestinal metabolite 5-hydroxytryptamine translocates via this axis, inducing bronchoconstriction and vasodilation (51). Short-chain fatty acids, primarily regulated by the gut microbiota, can translocate via the gut-lung axis by upregulating intestinal monocarboxylate transporters (51). This process alleviates Th2-mediated inflammatory responses and reduces IgE levels (51). Additionally, sodium benzoate intake reduced the proportion of anti-inflammatory gut bacteria, such as *Clostridium tyrobutyricum* and *Lactobacillus paracasei*, while increasing the abundance of pro-inflammatory species, including *Bacteroides thetaiotaomicron* and *Enterococcus faecalis* (52). Alterations in the gut microbiota lead to impaired intestinal barrier integrity, which may promote the translocation of inflammatory mediators via the gut-lung axis, subsequently altering the immune status of the respiratory system and contributing to the pathogenesis of asthma (53).

There are some limitations in this study. First, the sample was recruited exclusively from Nanjing, which might have limited the generalizability of the findings. Second, confounding variables such as BMI and parental smoking were not incorporated into the regression equations and mediation models, which may have reduced the reliability of the epidemiological conclusions. Third, the categories of food additives were limited in this study, which might have led to the exclusion of other food additives relevant to asthma associations. Finally, although this study explored the correlative links between food additive exposure, metabolome alterations, shifts in immune cell proportions, and asthma, it still lacks direct evidence demonstrating that food additives influence immune cell differentiation through metabolic pathways to ultimately induce asthma. To address these limitations, future investigations should incorporate multi-center populations and a more comprehensive range of food additives. More confounding variables should be considered. The effects of the key metabolites identified in this study should be directly applied both to animal and cellular models to elucidate the direct immune-related mechanisms through which these metabolites mediate food additive-induced asthma in future study.

## 5 Conclusion

In summary, food additives can exacerbate childhood asthma by immune-metabolism. This effect may be attributable to the ability of food additives to modulate the levels of key metabolites, including glycerophospholipids, fatty acyls, amino acids, amines, sphingolipids, sphingomyelins, and nucleotides. Our study highlights the significant impact of these changes in key metabolites on the proportions of helper T cells, and DCs, leading to a disruption of immune tolerance. Our findings provided valuable insights for preventing and treating childhood asthma and offer a scientific basis for improving food safety standards.

## Data Availability

The raw data supporting the conclusions of this article will be made available by the authors, without undue reservation.

## References

[1] Saltmarsh M. Food Additives and Why They Are Used. In: Saltmarsh M, editor. Saltmarsh's Essential Guide to Food Additives. United Kingdom: The Royal Society of Chemistry (2020).

[2] Marino M Puppo F Del Bo C Vinelli V Riso P Porrini M. A systematic review of worldwide consumption of ultra-processed foods: findings and criticisms. Nutrients. (2021) 13:2778. doi: 10.3390/nu13082778, PMID: 34444936 PMC8398521

[3] Goyal S Gupta M Sharma P Beniwal V. Hypersensitivity Associated with Food Additives. In: Nadda AK Goel G, editors. Microbes for Natural Food Additives. Singapore: Springer Nature Singapore (2022) 205–27.

[4] Martyn DM McNulty BA Nugent AP Gibney MJ. Food additives and preschool children. Proc Nutr Society. (2013) 72:109–16. doi: 10.1017/S0029665112002935, PMID: 23336563

[5] Trasande L Shaffer RM Sathyanarayana S. Food additives and child health. Pediatrics. (2018) 142:e20181410. doi: 10.1542/peds.2018-1410, PMID: 30037972 PMC6298598

[6] de Oliveira ZB Silva da Costa DV da Silva Dos Santos AC da Silva Júnior AQ de Lima Silva A de Santana RCF. Synthetic colors in food: A warning for children's health. Int J Environ Res Public Health. (2024) 21:682. doi: 10.3390/ijerph21060682, PMID: 38928929 PMC11203549

[7] Maslova E Strøm M Olsen SF Halldorsson TI. Consumption of artificially-sweetened soft drinks in pregnancy and risk of child asthma and allergic rhinitis. PloS One. (2013) 8:e57261. doi: 10.1371/journal.pone.0057261, PMID: 23460835 PMC3584110

[8] Qurrat ul A Khan SA. Artificial sweeteners: safe or unsafe? J Pak Med Assoc. (2015) 65:225–7. 25842566

[9] Lemoine A Pauliat-Desbordes S Challier P Tounian P. Adverse reactions to food additives in children: A retrospective study and a prospective survey. Arch Pediatr. (2020) 27:368–71. doi: 10.1016/j.arcped.2020.07.005, PMID: 32807620

[10] Quirós-Alcalá L Hansel NN McCormack MC Matsui EC. Paraben exposures and asthma-related outcomes among children from the US general population. J Allergy Clin Immunol. (2019) 143:948–56.e4. doi: 10.1016/j.jaci.2018.08.021, PMID: 30194988 PMC6691726

[11] Leo L Loong C Ho XL Raman MFB Suan MYT Loke WM. Occurrence of azo food dyes and their effects on cellular inflammatory responses. Nutrition. (2018) 46:36–40. doi: 10.1016/j.nut.2017.08.010, PMID: 29290353

[12] Yamashita H Matsuhara H Miotani S Sako Y Matsui T Tanaka H. Artificial sweeteners and mixture of food additives cause to break oral tolerance and induce food allergy in murine oral tolerance model for food allergy. Clin Exp Allergy. (2017) 47:1204–13. doi: 10.1111/cea.2017.47.issue-9, PMID: 28370609

[13] Dutta S Sengupta P. Men and mice: Relating their ages. Life Sci. (2016) 152:244–8. doi: 10.1016/j.lfs.2015.10.025, PMID: 26596563

[14] Daubeuf F Frossard N. Acute asthma models to ovalbumin in the mouse. Curr Protoc Mouse Biol. (2013) 3:31–7. doi: 10.1002/9780470942390.2013.3.issue-1 26069022

[15] Van Loco J Vandevijvere S Cimenci O Vinkx C Goscinny S. Dietary exposure of the Belgian adult population to 70 food additives with numerical ADI. Food Control. (2015) 54:86–94. doi: 10.1016/j.foodcont.2015.01.029

[16] Schmidt U Weigert M Broaddus C Myers G eds. Cell Detection with Star-Convex Polygons. Cham: Springer International Publishing (2018).

[17] Weigert M Schmidt U Haase R Sugawara K Myers G eds. (2020). Star-convex polyhedra for 3D object detection and segmentation in microscopy, in: 2020 IEEE Winter Conference on Applications of Computer Vision (WACV), Snowmass, CO, USA, pp. 1–5.

[18] Weigert M Schmidt U eds. (2022). Nuclei instance segmentation and classification in histopathology images with stardist, in: 2022 IEEE International Symposium on Biomedical Imaging Challenges (ISBIC), Kolkata, India, pp. 28–31.

[19] Kumar N Verma R Sharma S Bhargava S Vahadane A Sethi A. A dataset and a technique for generalized nuclear segmentation for computational pathology. IEEE Trans Med Imaging. (2017) 36:1550–60. doi: 10.1109/TMI.2017.2677499, PMID: 28287963

[20] Naylor P Laé M Reyal F Walter T. Segmentation of nuclei in histopathology images by deep regression of the distance map. IEEE Trans Med Imaging. (2019) 38:448–59. doi: 10.1109/TMI.2018.2865709, PMID: 30716022

[21] You L Kou J Wang M Ji G Li X Su C. An exposome atlas of serum reveals the risk of chronic diseases in the Chinese population. Nat Commun. (2024) 15:2268. doi: 10.1038/s41467-024-46595-z, PMID: 38480749 PMC10937660

[22] Dmitrienko SG Kochuk EV Apyari VV Tolmacheva VV Zolotov YA. Recent advances in sample preparation techniques and methods of sulfonamides detection – A review. Analytica Chimica Acta. (2014) 850:6–25. doi: 10.1016/j.aca.2014.08.023, PMID: 25441155

[23] Yongfei Y Huihua C PingGu W. Determination of veterinary drug residues by liquid chromatography-tandem mass spectrometry. J Analytical Sci. (2008) 2008:359–66.

[24] Bülbül G Celli GB Zaferani M Raghupathi K Galopin C Abbaspourrad A. Quantitative comparison of adsorption and desorption of commonly used sweeteners in the oral cavity. Food Chem. (2019) 271:577–80. doi: 10.1016/j.foodchem.2018.07.221, PMID: 30236718

[25] Oktavirina V Prabawati NB Fathimah RN Palma M Kurnia KA Darmawan N. Analytical methods for determination of non-nutritive sweeteners in foodstuffs. Molecules. (2021) 26:3135. doi: 10.3390/molecules26113135, PMID: 34073913 PMC8197393

[26] Montera V Martins APB Borges CA Canella DS. Distribution and patterns of use of food additives in foods and beverages available in Brazilian supermarkets. Food Funct. (2021) 12:7699–708. doi: 10.1039/D1FO00429H, PMID: 34282819

[27] Additives EPoF, Food NSat. Scientific Opinion on the re-evaluation of aspartame (E 951) as a food additive. EFSA J. (2013) 11:3496. doi: 10.2903/j.efsa.2013.3496

[28] Weiss A. Hydrophilic drug delivery based on gelatin nanoparticles. Saarland University. (2018).

[29] Magnuson BA Roberts A Nestmann ER. Critical review of the current literature on the safety of sucralose. Food Chem Toxicol. (2017) 106:324–55. doi: 10.1016/j.fct.2017.05.047, PMID: 28558975

[30] Gómez-Fernández AR Santacruz A Jacobo-Velázquez DA. The complex relationship between metabolic syndrome and sweeteners. J Food Sci. (2021) 86:1511–31. doi: 10.1111/1750-3841.15709, PMID: 33908634

[31] Genton C Frei PC Pécoud A. Value of oral provocation tests to aspirin and food additives in the routine investigation of asthma and chronic urticaria. J Allergy Clin Immunol. (1985) 76:40–5. doi: 10.1016/0091-6749(85)90802-4, PMID: 2861222

[32] Şengul Emeksiz Z Özmen S. The case of a child with contact urticaria due to sodium benzoate treatment. Contact Dermatitis. (2022) 86:40–1. doi: 10.1111/cod.13916, PMID: 34137041

[33] Morianos I Semitekolou M. Dendritic cells: critical regulators of allergic asthma. Int J Mol Sci. (2020) 21:7930. doi: 10.3390/ijms21217930, PMID: 33114551 PMC7663753

[34] Chen W Cao Y Zhong Y Sun J Dong J. The mechanisms of effector th cell responses contribute to treg cell function: new insights into pathogenesis and therapy of asthma. Front Immunol. (2022) 13:862866. doi: 10.3389/fimmu.2022.862866, PMID: 35898499 PMC9309477

[35] Maier E Kurz K Jenny M Schennach H Ueberall F Fuchs D. Food preservatives sodium benzoate and propionic acid and colorant curcumin suppress Th1-type immune response *in vitro* . Food Chem Toxicol. (2010) 48:1950–6. doi: 10.1016/j.fct.2010.04.042, PMID: 20435078

[36] Yeroshenko G Donets I Shevchenko K Riabushko O Zviaholska I. The impact of food additives complex on the structural organization of pulmonary diffuse lymphoid tissue shown in the experiment. World Med. Biol. (2023) 4:193–7. doi: 10.26724/2079-8334-2023-4-86-193-197

[37] Wang Z Yang M Yang Y He Y Qian H. Structural basis for catalysis of human choline/ethanolamine phosphotransferase 1. Nat Commun. (2023) 14:2529. doi: 10.1038/s41467-023-38290-2, PMID: 37137909 PMC10156783

[38] Zhao B Shi C Wang X Sun Z Ruan Y Wang X. Kechuan Decoction mitigates apoptosis of airway epithelial cells by improving lipid metabolism disorders and mitochondria dysfunction in HDM-induced asthma. Phytomedicine. (2025) 136:156299. doi: 10.1016/j.phymed.2024.156299, PMID: 39671785

[39] Yuehua J Jianqiao C Xiao L Ping J Chuanhua Y. Biological function of phosphatidylcholine and its role in cardiovascular diseases. Chem Life. (2022) 42:412–23. doi: 10.13488/j.smhx.20210870

[40] Qin X Qiu C Zhao L. Lysophosphatidylcholine perpetuates macrophage polarization toward classically activated phenotype in inflammation. Cell Immunol. (2014) 289:185–90. doi: 10.1016/j.cellimm.2014.04.010, PMID: 24841857

[41] Wang S Tang K Lu Y Tian Z Huang Z Wang M. Revealing the role of glycerophospholipid metabolism in asthma through plasma lipidomics. Clinica Chimica Acta. (2021) 513:34–42. doi: 10.1016/j.cca.2020.11.026, PMID: 33307061

[42] Chen M Su Q Shi Y. Molecular mechanism of IgE-mediated FcϵRI activation. Nature. (2025) 637:453–60. doi: 10.1038/s41586-024-08229-8, PMID: 39442557

[43] Zhu Z Camargo CA Raita Y Fujiogi M Liang L Rhee EP. Metabolome subtyping of severe bronchiolitis in infancy and risk of childhood asthma. J Allergy Clin Immunol. (2022) 149:102–12. doi: 10.1016/j.jaci.2021.05.036, PMID: 34119532 PMC8660920

[44] Saluja R Kumar A Jain M Goel SK Jain A. Role of sphingosine-1-phosphate in mast cell functions and asthma and its regulation by non-coding RNA. Front Immunol. (2017) 8:587. doi: 10.3389/fimmu.2017.00587, PMID: 28588581 PMC5439123

[45] Patel KR Bai Y Trieu KG Barrios J Ai X. Targeting acetylcholine receptor M3 prevents the progression of airway hyperreactivity in a mouse model of childhood asthma. FASEB J. (2017) 31:4335–46. doi: 10.1096/fj.201700186R, PMID: 28619712 PMC5602904

[46] Wu W Samoszuk MK Comhair SA Thomassen MJ Farver CF Dweik RA. Eosinophils generate brominating oxidants in allergen-induced asthma. J Clin Invest. (2000) 105:1455–63. doi: 10.1172/JCI9702, PMID: 10811853 PMC315470

[47] Gao H Sun Z Xiao C Zheng X Zhang Y. The metabonomic study of Shaoyao-Gancao decoction in a rat model of acute bronchial asthma by 1H NMR. Analytical Methods. (2016) 8:570–81. doi: 10.1039/C5AY01701G

[48] Shil A Olusanya O Ghufoor Z Forson B Marks J Chichger H. Artificial sweeteners disrupt tight junctions and barrier function in the intestinal epithelium through activation of the sweet taste receptor, T1R3. Nutrients. (2020) 12:1862. doi: 10.3390/nu12061862, PMID: 32580504 PMC7353258

[49] Dickson RP Singer BH Newstead MW Falkowski NR Erb-Downward JR Standiford TJ. Enrichment of the lung microbiome with gut bacteria in sepsis and the acute respiratory distress syndrome. Nat Microbiol. (2016) 1:16113. doi: 10.1038/nmicrobiol.2016.113, PMID: 27670109 PMC5076472

[50] Bradley CP Teng F Felix KM Sano T Naskar D Block KE. Segmented filamentous bacteria provoke lung autoimmunity by inducing gut-lung axis th17 cells expressing dual TCRs. Cell Host Microbe. (2017) 22:697–704.e4. doi: 10.1016/j.chom.2017.10.007, PMID: 29120746 PMC5749641

[51] Guo HH Han YX Rong XJ Shen Z Shen HR Kong LF. Alleviation of allergic asthma by rosmarinic acid via gut-lung axis. Phytomedicine. (2024) 126:155470. doi: 10.1016/j.phymed.2024.155470, PMID: 38417242

[52] Hrncirova L Hudcovic T Sukova E Machova V Trckova E Krejsek J. Human gut microbes are susceptible to antimicrobial food additives *in vitro* . Folia Microbiol (Praha). (2019) 64:497–508. doi: 10.1007/s12223-018-00674-z, PMID: 30656592

[53] Steck N Hoffmann M Sava IG Kim SC Hahne H Tonkonogy SL. Enterococcus faecalis metalloprotease compromises epithelial barrier and contributes to intestinal inflammation. Gastroenterology. (2011) 141:959–71. doi: 10.1053/j.gastro.2011.05.035, PMID: 21699778

