# Supplementary material for: The study of the relationship between food additives and the childhood asthma based on metabolome analysis
Source: Front Immunol. 2025 Sep 26;16:1671022. doi: 10.3389/fimmu.2025.1671022 (PMC12510829; doi:10.3389/fimmu.2025.1671022)
Supplement: Supplementary file 1 [file DataSheet1.docx]

Supplementary Material

# **Supplementary Figures and Tables**

- 1. **Supplementary Tables**

**Supplementary Table 1.** The UPLC gradient elution program for food additives.

| **Time windows (min)** | **Concentrations of phase B (%)** |
| --- | --- |
| **0.0-1.0** | 5 |
| **1.0-6.0** | 5-65 |
| **6.0-7.0** | 65-95 |
| **7.0-9.0** | 95 |
| **9.0-9.1** | 95-5 |
| **9.1-11** | 5 |

**Supplementary Table 2.** The mass spectrometry parameters for food additives.

| **Food additives** | **Ion mode** | **Retention time (min)** | **Precursor ions (m/z)** | **Dwell time (ms)** | **Spray ion voltage (kV)** | **Ion source temperature (℃)** | **collision gas (psi)** | **curtain gas (psi)** | **nebulizing gas (psi)** | **drying gas (psi)** | **Product ions (m/z)** | **Declustering potential (V)** | **Collision energy (V)** |
| --- | --- | --- | --- | --- | --- | --- | --- | --- | --- | --- | --- | --- | --- |
| Sodium saccharin | [M-Na]^-^ | 3.41 | 182 | 100 | -4.5 | 550 | 50 | 50 | 50 | 50 | 42 | -80 | -54 |
|  |  |  |  |  |  |  |  |  |  |  | 105.9* | -80 | -20 |
| Cyclamate | [M-Na]^-^ | 3.81 | 178 | 100 | -4.5 | 550 | 50 | 50 | 50 | 50 | 79.9 | -80 | -37 |
|  |  |  |  |  |  |  |  |  |  |  | 95.9* | -80 | -28 |
| Acesulfame | [M-K]^-^ | 2.32 | 161.8 | 100 | -4.5 | 550 | 50 | 50 | 50 | 50 | 81.7 | -80 | -40 |
|  |  |  |  |  |  |  |  |  |  |  | 77.8* | -80 | -17 |
| Sucralose | [M-H]^-^ | 4.86 | 395.1 | 100 | -4.5 | 550 | 50 | 50 | 50 | 50 | 395.1 | -80 | -10 |
|  |  |  |  |  |  |  |  |  |  |  | 34.9* | -80 | -25 |
| Neotame | [M-H]^-^ | 6.30 | 377.2 | 100 | -4.5 | 550 | 50 | 50 | 50 | 50 | 200.2 | -80 | -25 |
|  |  |  |  |  |  |  |  |  |  |  | 301.2* | -80 | -25 |
| Aspartame | [M-H]^-^ | 4.77 | 293 | 100 | -4.5 | 550 | 50 | 50 | 50 | 50 | 261 | -80 | -23 |
|  |  |  |  |  |  |  |  |  |  |  | 200* | -80 | -14 |
| Benzoic acid | [M-H]^-^ | 2.32 | 121 | 100 | -4.5 | 550 | 50 | 50 | 50 | 50 | 77 | -36 | -18 |
| Dehydroacetic acid | [M-H]^-^ | 2.98 | 167 | 100 | -4.5 | 550 | 50 | 50 | 50 | 50 | 82.9 | -47 | -17 |
|  |  |  |  |  |  |  |  |  |  |  | 123* | -47 | -10 |
| Ponceau 4R | [M-2Na]^2-^ | 3.81 | 268 | 100 | -4.5 | 550 | 50 | 50 | 50 | 50 | 205.9 | -72 | -15 |
|  |  |  |  |  |  |  |  |  |  |  | 79.8* | -72 | -67 |
| Sunset yellow | [M-2Na]^2-^ | 4.20 | 203 | 100 | -4.5 | 550 | 50 | 50 | 50 | 50 | 170.9 | -50 | -19 |
|  |  |  |  |  |  |  |  |  |  |  | 155.8* | -50 | -25 |

*: Qualifier ions

**Supplementary Table 3.** The UPLC gradient elution program for untargeted metabolome.

| **Time windows (min)** | **Concentrations of phase B (%)** |
| --- | --- |
| 0-3 | 99 |
| 3-10 | 99-1 |
| 10-15 | 1 |
| 15-15.1 | 1-99 |
| 15.1-17 | 99 |

**Supplementary Table 4.** The mass spectrometry parameters for untargeted metabolome.

| **Parameters** | **Values** |
| --- | --- |
| Capillary voltage | +3.5 kV |
| sheath gas | 35 arbitrary unit |
| Auxiliary gas | 10 arbitrary unit |
| Flow rate of dry gas | 10.0 L/min |
| Atomization temperature | 350℃ |
| Capillary temperature | 263℃ |
| Full MS scan range | 100-1200 m/z |

**Supplementary Table 5.** The characteristics of the subjects in this study.

| **Variables** | **Values** | |  |
| --- | --- | --- | --- |
| **Gender** | **Asthmatic group** | **Control group** | ***P*** |
| Male | 73 | 58 | 0.052 |
| Female | 47 | 62 |  |
| **Age** |  |  |  |
| <1 | 0 | 4 | 0.285 |
| 1-6 | 102 | 86 |  |
| >6 | 18 | 30 |  |

**Supplementary Table 6.** Asthma-related metabolites in the serum.

| **Categories** | **Names** | **HMDB IDs** |
| --- | --- | --- |
| Amines | Dioctylamine | HMDB0251428 |
|  | Spermidine | HMDB0001257 |
|  | Spermine | HMDB0001256 |
|  | Homocystamine | HMDB0253204 |
| Amino acids | 4-Oxo-L-proline | HMDB0246561 |
|  | Arginylhistidine | HMDB0028711 |
|  | Arginylleucine | HMDB0028713 |
|  | Glutamic acid | HMDB0000148 |
|  | Glutamine | HMDB0000641 |
|  | Histidine | HMDB0000177 |
|  | L-Cystine | HMDB0000192 |
|  | Leucine | HMDB0000687 |
|  | Leucylalanine | HMDB0028922 |
|  | L-Pipecolic acid | HMDB0000716 |
|  | N-Acryloylglycine | HMDB0001843 |
|  | N-Lactoylphenylalanine | HMDB0062175 |
|  | Phenylalanylleucine | HMDB0253025 |
|  | Serylleucine | HMDB0029043 |
|  | Kynurenine | HMDB0000684 |
|  | Palmitic amide | HMDB0012273 |
|  | Palmitoleamide | HMDB0256086 |
| Fatty acyls | 2-Hexenoylcarnitine | HMDB0013161 |
|  | 3-Decenoylcarnitine | HMDB0241067 |
|  | 6-Octenoylcarnitine | HMDB0241700 |
|  | Hexanoylcarnitine | HMDB0000756 |
|  | Isobutyryl-L-carnitine | HMDB0000736 |
|  | L-Acetylcarnitine | HMDB0000201 |
|  | Sebacoyl-L-carnitine | HMDB0240726 |
|  | hepta-2,5-dienoic acid | HMDB0340968 |
|  | non-3-enedioic acid | HMDB0341022 |
|  | Sebacic acid | HMDB0000792 |
|  | Undecanedioic acid | HMDB0000888 |
|  | Acetylcholine | HMDB0000895 |
|  | Vitamin A | HMDB0000305 |
| Glycerolipids | DG(PGF1alpha/0:0/2:0) | HMDB0297050 |
| Glycerophospholipids | LysoPA(i-12:0/0:0) | HMDB0114763 |
|  | PA(8:0/12:0) | HMDB0115483 |
|  | PA(i-21:0/8:0) | HMDB0115872 |
|  | PA(LTE4/13:0) | HMDB0262885 |
|  | PA(PGE2/P-16:0) | HMDB0266716 |
|  | PA(TXB2/i-17:0) | HMDB0267870 |
|  | Glycerophosphocholine | HMDB0000086 |
|  | LysoPC(17:0/0:0) | HMDB0012108 |
|  | LysoPC(28:0/0:0) | HMDB0029206 |
|  | PC(14:0/14:0) | HMDB0007866 |
|  | PC(18:1(9Z)e/2:0) | HMDB0011148 |
|  | PC(36:0) | HMDB0007886 |
|  | LysoPE(14:0/0:0) | HMDB0011500 |
|  | LysoPE(14:1(9Z)/0:0) | HMDB0011501 |
|  | LysoPE(P-18:0/0:0) | HMDB0240598 |
|  | PE(18:2(9Z,12Z)/14:0) | HMDB0009085 |
|  | PE(TXB2/15:0) | HMDB0260817 |
|  | PGP(i-14:0/i-17:0) | HMDB0116574 |
|  | PA(TXB2/i-15:0) | HMDB0267662 |
|  | PS(5-iso PGF2VI/14:0) | HMDB0280844 |
|  | PS(PGJ2/14:1(9Z)) | HMDB0280935 |
|  | GlcCer(d18:1/16:0) | HMDB0004971 |
| Indoles | Indole | HMDB0000738 |
|  | Indoleacetaldehyde | HMDB0001190 |
| Nucleosides | Hypoxanthine | HMDB0000157 |
|  | 5-Methylcytidine | HMDB0000982 |
| Organosulfonic acids and derivatives | N-Ornithyl-L-taurine | HMDB0033519 |
|  | Taurine | HMDB0000251 |
| Phosphosphingolipids | LysoSM(d18:1) | HMDB0006482 |
|  | Sphingosine-1-phosphate | HMDB0000277 |
| Sphingolipids | sphinganine (C20) | HMDB0304489 |
|  | Hexadecasphingosine | HMDB0242181 |
|  | Sphingosine | HMDB0000252 |
|  | C16-Ceramide | HMDB0249508 |
|  | Cer(d18:2/18:1) | HMDB0341547 |
|  | Cer(d18:2/20:0) | HMDB0341548 |
|  | Cer(d20:1/PGD2) | HMDB0290110 |
| Sterol Lipids | Sterol | HMDB0060512 |

**Supplementary Table 7.** Mediation analysis of differential metabolites which played a mediating role in the relationship between food additives and childhood asthma.

| **FAs → Metabolites** | **Total Effect** | ***P*** | **Indirect Effect** | ***P*** | **Direct Effect** | ***P*** |
| --- | --- | --- | --- | --- | --- | --- |
| Benzoic Acid → sphinganine (C20) | 0.00057328 (0.00038080, 0.00076052) | 0 | 0.00013741 (0.00004777, 0.00022238) | 0.004 | 0.00043587 (0.00024743, 0.00063255) | 0 |
| Benzoic Acid → N-Acryloylglycine | 0.00047913 (0.00029950, 0.00064969) | 0 | 0.00018452 (0.00008411, 0.00028945) | 0.002 | 0.00029460 (0.00012693, 0.00046313) | 0.002 |
| Benzoic Acid → PE(P-16:0/PGE1) | 0.00058112 (0.00044283, 0.00070766) | 0 | 0.00020225 (0.00010651, 0.00030691) | 0 | 0.00037887 (0.00024383, 0.00052227) | 0 |
| Benzoic Acid → PGP(i-14:0/i-17:0) | 0.00064773 (0.00026841, 0.00105713) | 0 | 0.00012613 (0.00003839, 0.00021021) | 0.002 | 0.00052160 (0.00011866, 0.00091306) | 0.008 |
| Benzoic Acid → PS(PGJ2/14:1(9Z)) | 0.00039666 (0.00015686, 0.00061644) | 0 | 0.00030060 (0.00018493, 0.00042418) | 0 | 0.00009606 (-0.00013228, 0.00033058) | 0.422 |
| Benzoic Acid → PS(5-iso PGF2VI/14:0) | 0.00042408 (0.00020425, 0.00063422) | 0 | 0.00027025 (0.00015856, 0.00038014) | 0 | 0.00015384 (-0.00005961, 0.00037113) | 0.156 |
| Benzoic Acid → GlcCer(d18:1/16:0) | 0.00065538 (0.00049862, 0.00079518) | 0 | 0.00007751 (0.00000523, 0.00014908) | 0.04 | 0.00057787 (0.00042774, 0.00072216) | 0 |
| Benzoic Acid → Sterol | 0.00053146 (0.00036386, 0.00068896) | 0 | 0.00021194 (0.00010487, 0.00032414) | 0 | 0.00031952 (0.00016071, 0.00047481) | 0 |
| Benzoic Acid → Leucine | 0.00060360 (0.00028936, 0.00095617) | 0 | 0.00013719 (0.00005496, 0.00022435) | 0 | 0.00046641 (0.00015880, 0.00081959) | 0.002 |
| Benzoic Acid → Taurine | 0.00059842 (0.00028676, 0.00093243) | 0 | 0.00019559 (0.00010491, 0.00029527) | 0 | 0.00040283 (0.00009181, 0.00073763) | 0.006 |
| Benzoic Acid → N-Ornithyl-L-taurine | 0.00071335 (0.00014170, 0.00132654) | 0.014 | 0.00004480 (0.00000678, 0.00009699) | 0.018 | 0.00066855 (0.00009448, 0.00126527) | 0.026 |
| Benzoic Acid → Sphingosine-1-phosphate | 0.00042210 (0.00025033, 0.00062272) | 0.002 | 0.00025952 (0.00013928, 0.00040229) | 0 | 0.00016258 (0.00001499, 0.00032212) | 0.038 |
| Benzoic Acid → Hypoxanthine | 0.00067370 (0.00048694, 0.00087995) | 0 | 0.00010389 (0.00002832, 0.00018619) | 0.016 | 0.00056981 (0.00040274, 0.00076178) | 0 |
| Benzoic Acid → 5-Methylcytidine | 0.00079078 (0.00059224, 0.00100852) | 0 | 0.00008686 (0.00002609, 0.00015747) | 0.004 | 0.00070392 (0.00049379, 0.00094189) | 0 |
| Benzoic Acid → Glutamine | 0.00058555 (0.00040920, 0.00075373) | 0 | 0.00010767 (0.00001716, 0.00019747) | 0.026 | 0.00047788 (0.00031334, 0.00065695) | 0 |
| Benzoic Acid → Acetylcholine | 0.00058337 (0.00042495, 0.00075732) | 0 | 0.00017381 (0.00008444, 0.00026834) | 0 | 0.00040956 (0.00024882, 0.00058310) | 0 |
| Benzoic Acid → Glutamic acid | 0.00045395 (0.00026917, 0.00063745) | 0 | 0.00026777 (0.00016447, 0.00037895) | 0 | 0.00018618 (0.00000529, 0.00034597) | 0.044 |
| Benzoic Acid → Histidine | 0.00067464 (0.00050159, 0.00083673) | 0 | 0.00009366 (0.00001903, 0.00017399) | 0.018 | 0.00058097 (0.00040355, 0.00075570) | 0 |
| Benzoic Acid → L-Cystine | 0.00053928 (0.00036559, 0.00068596) | 0 | 0.00013096 (0.00004488, 0.00022535) | 0.004 | 0.00040831 (0.00023002, 0.00056819) | 0 |
| Benzoic Acid → Phenylalanylleucine | 0.00066680 (0.00037711, 0.00093463) | 0.004 | 0.00032149 (0.00019014, 0.00048226) | 0.002 | 0.00034531 (0.00005905, 0.00056166) | 0.028 |
| Benzoic Acid → Arginylleucine | 0.00065162 (0.00044481, 0.00085825) | 0 | 0.00008339 (0.00001281, 0.00016594) | 0.028 | 0.00056823 (0.00034233, 0.00078332) | 0 |
| Benzoic Acid → Arginylhistidine | 0.00071803 (0.00061427, 0.00078178) | 0 | 0.00004646 (0.00001095, 0.00009572) | 0.006 | 0.00067158 (0.00057347, 0.00074670) | 0 |
| Benzoic Acid → N-Lactoylphenylalanine | 0.00071916 (0.00046993, 0.00092008) | 0 | 0.00024249 (0.00013048, 0.00036173) | 0 | 0.00047667 (0.00022501, 0.00070407) | 0.002 |
| Benzoic Acid → Spermidine | 0.00051067 (0.00030461, 0.00075719) | 0 | 0.00015018 (0.00006521, 0.00024451) | 0 | 0.00036049 (0.00016654, 0.00059919) | 0 |
| Benzoic Acid → C16-Ceramide | 0.00057357 (0.00045432, 0.00070266) | 0 | 0.00013075 (0.00004872, 0.00021262) | 0.004 | 0.00044282 (0.00032535, 0.00056719) | 0 |
| Benzoic Acid → Spermine | 0.00044412 (0.00027869, 0.00062056) | 0 | 0.00012926 (0.00003015, 0.00022864) | 0.002 | 0.00031485 (0.00016938, 0.00046771) | 0 |
| Benzoic Acid → Cer(d18:2/20:0) | 0.00069867 (0.00059843, 0.00079347) | 0 | 0.00007823 (0.00002138, 0.00014646) | 0.006 | 0.00062044 (0.00050558, 0.00072729) | 0 |
| Benzoic Acid → Cer(d20:1/PGD2) | 0.00033832 (0.00019959, 0.00048189) | 0 | 0.00033291 (0.00021359, 0.00045800) | 0 | 0.00000541 (-0.00009176, 0.00010456) | 0.93 |
| Benzoic Acid → Dioctylamine | 0.00058452 (0.00041609, 0.00074048) | 0 | 0.00018845 (0.00009649, 0.00028611) | 0 | 0.00039608 (0.00021471, 0.00057728) | 0 |
| Benzoic Acid → Homocystamine | 0.00046871 (0.00013633, 0.00080629) | 0.016 | 0.00025890 (0.00014352, 0.00039457) | 0 | 0.00020981 (-0.00012999, 0.00053340) | 0.2 |
| Benzoic Acid → Hexanoylcarnitine | 0.00047153 (0.00029032, 0.00070073) | 0 | 0.00015008 (0.00005472, 0.00026768) | 0.01 | 0.00032145 (0.00019120, 0.00047727) | 0 |
| Benzoic Acid → 6-Octenoylcarnitine | 0.00069401 (0.00040209, 0.00103825) | 0 | 0.00024894 (0.00015648, 0.00036233) | 0 | 0.00044508 (0.00017278, 0.00076294) | 0.008 |
| Benzoic Acid → Hexadecasphingosine | 0.00047348 (0.00018864, 0.00074300) | 0 | 0.00017700 (0.00007808, 0.00027927) | 0 | 0.00029648 (0.00003653, 0.00056616) | 0.022 |
| Benzoic Acid → Isobutyryl-L-carnitine | 0.00062335 (0.00037742, 0.00088704) | 0 | 0.00020948 (0.00012109, 0.00030411) | 0 | 0.00041387 (0.00014690, 0.00069364) | 0.008 |
| Benzoic Acid → 3-Decenoylcarnitine | 0.00043450 (0.00004879, 0.00077752) | 0.028 | 0.00030769 (0.00017850, 0.00045087) | 0 | 0.00012681 (-0.00025113, 0.00048213) | 0.47 |
| Benzoic Acid → 2-Hexenoylcarnitine | 0.00045641 (0.00027440, 0.00063436) | 0 | 0.00016270 (0.00005351, 0.00026945) | 0.002 | 0.00029370 (0.00013992, 0.00045723) | 0 |
| Benzoic Acid → L-Acetylcarnitine | 0.00076453 (0.00058622, 0.00090763) | 0 | 0.00019118 (0.00009937, 0.00029084) | 0 | 0.00057334 (0.00036557, 0.00073715) | 0 |
| Benzoic Acid → hepta-2,5-dienoic acid | 0.00072840 (0.00054343, 0.00090464) | 0 | 0.00005853 (0.00000572, 0.00012048) | 0.042 | 0.00066987 (0.00047214, 0.00085233) | 0 |
| Benzoic Acid → Sphingosine | 0.00069460 (0.00053283, 0.00086103) | 0 | 0.00010308 (0.00002822, 0.00017994) | 0.008 | 0.00059152 (0.00044031, 0.00075567) | 0 |
| Benzoic Acid → Palmitic amide | 0.00048912 (0.00031314, 0.00066784) | 0 | 0.00018718 (0.00008925, 0.00029096) | 0 | 0.00030194 (0.00014175, 0.00048472) | 0 |
| Benzoic Acid → Palmitoleamide | 0.00052795 (0.00038078, 0.00067709) | 0 | 0.00012516 (0.00002826, 0.00021985) | 0.024 | 0.00040280 (0.00025795, 0.00054389) | 0 |
| Benzoic Acid → 4-Oxo-L-proline | 0.00045908 (0.00028974, 0.00066100) | 0 | 0.00016924 (0.00007748, 0.00027305) | 0 | 0.00028984 (0.00014318, 0.00046892) | 0 |
| Benzoic Acid → PA(i-21:0/8:0) | 0.00078992 (0.00061291, 0.00097906) | 0 | 0.00062555 (0.00043477, 0.00082610) | 0 | 0.00016437 (0.00003818, 0.00028094) | 0.014 |
| Benzoic Acid → PA(TXB2/i-17:0) | 0.00054859 (0.00035987, 0.00072752) | 0 | 0.00026402 (0.00016661, 0.00037461) | 0 | 0.00028457 (0.00009527, 0.00046594) | 0.006 |
| Benzoic Acid → PA(LTE4/13:0) | 0.00047783 (0.00016785, 0.00079908) | 0.006 | 0.00028010 (0.00017196, 0.00040423) | 0 | 0.00019773 (-0.00012499, 0.00051978) | 0.21 |
| Benzoic Acid → LysoPC(28:0/0:0) | 0.00038045 (0.00018713, 0.00058018) | 0 | 0.00028498 (0.00017514, 0.00040436) | 0 | 0.00009547 (-0.00008398, 0.00027188) | 0.31 |
| Benzoic Acid → Glycerophosphocholine | 0.00052065 (0.00039017, 0.00066342) | 0 | 0.00022598 (0.00012846, 0.00033021) | 0 | 0.00029467 (0.00018789, 0.00041098) | 0 |
| Benzoic Acid → PC(18:1(9Z)e/2:0) | 0.00071144 (0.00049425, 0.00090979) | 0 | 0.00021040 (0.00011492, 0.00031945) | 0 | 0.00050104 (0.00027825, 0.00069071) | 0 |
| Benzoic Acid → PC(14:0/14:0) | 0.00059703 (0.00036447, 0.00081918) | 0 | 0.00014662 (0.00005192, 0.00024017) | 0.004 | 0.00045041 (0.00023423, 0.00068215) | 0 |
| Benzoic Acid → LysoPE(P-18:0/0:0) | 0.00070597 (0.00060452, 0.00078817) | 0 | 0.00007479 (0.00002345, 0.00013925) | 0 | 0.00063118 (0.00052166, 0.00072694) | 0 |
| Benzoic Acid → PE(18:2(9Z,12Z)/14:0) | 0.00056242 (0.00040007, 0.00072776) | 0 | 0.00018651 (0.00008630, 0.00029668) | 0 | 0.00037591 (0.00023273, 0.00052761) | 0 |
| Dehydroacetic Acid → sphinganine (C20) | 0.00025514 (0.00018930, 0.00031901) | 0 | 0.00003638 (0.00001891, 0.00005621) | 0.002 | 0.00021876 (0.00015364, 0.00029011) | 0 |
| Dehydroacetic Acid → N-Acryloylglycine | 0.00022954 (0.00014045, 0.00032256) | 0 | 0.00004338 (0.00002142, 0.00006599) | 0 | 0.00018615 (0.00009316, 0.00027976) | 0 |
| Dehydroacetic Acid → PGP(i-14:0/i-17:0) | 0.00022460 (0.00002563, 0.00041552) | 0.024 | 0.00003911 (0.00002159, 0.00005841) | 0 | 0.00018549 (-0.00000994, 0.00037831) | 0.062 |
| Dehydroacetic Acid → PS(PGJ2/14:1(9Z)) | 0.00028594 (0.00012757, 0.00042639) | 0.004 | 0.00004682 (0.00002716, 0.00006793) | 0 | 0.00023912 (0.00007546, 0.00038019) | 0.01 |
| Dehydroacetic Acid → PS(5-iso PGF2VI/14:0) | 0.00023867 (0.00011447, 0.00036763) | 0 | 0.00004618 (0.00002684, 0.00006734) | 0 | 0.00019249 (0.00006652, 0.00032402) | 0.004 |
| Dehydroacetic Acid → GlcCer(d18:1/16:0) | 0.00032101 (0.00020554, 0.00042675) | 0 | 0.00002809 (0.00001374, 0.00004397) | 0 | 0.00029292 (0.00017935, 0.00039979) | 0 |
| Dehydroacetic Acid → L-Pipecolic acid | 0.00032136 (0.00023145, 0.00042320) | 0 | 0.00003778 (0.00001841, 0.00005729) | 0 | 0.00028358 (0.00019240, 0.00038876) | 0 |
| Dehydroacetic Acid → Sterol | 0.00032361 (0.00025556, 0.00039747) | 0 | 0.00003401 (0.00001441, 0.00005571) | 0.002 | 0.00028961 (0.00022557, 0.00035755) | 0 |
| Dehydroacetic Acid → Indole | 0.00034844 (0.00023349, 0.00046295) | 0 | 0.00002599 (0.00000943, 0.00004444) | 0 | 0.00032246 (0.00020853, 0.00043420) | 0 |
| Dehydroacetic Acid → Indoleacetaldehyde | 0.00038447 (0.00014255, 0.00063516) | 0.002 | 0.00001824 (0.00000722, 0.00003335) | 0.002 | 0.00036623 (0.00012433, 0.00061412) | 0.004 |
| Dehydroacetic Acid → Taurine | 0.00024961 (0.00017356, 0.00032554) | 0 | 0.00004814 (0.00002804, 0.00007072) | 0 | 0.00020147 (0.00012177, 0.00027779) | 0 |
| Dehydroacetic Acid → Hypoxanthine | 0.00023600 (0.00014547, 0.00032685) | 0 | 0.00004665 (0.00002685, 0.00006883) | 0 | 0.00018935 (0.00010214, 0.00027959) | 0 |
| Dehydroacetic Acid → 5-Methylcytidine | 0.00036226 (0.00028801, 0.00041580) | 0 | 0.00001817 (0.00000430, 0.00003323) | 0.014 | 0.00034409 (0.00026815, 0.00040414) | 0 |
| Dehydroacetic Acid → Glutamine | 0.00028397 (0.00016823, 0.00041703) | 0 | 0.00003148 (0.00001323, 0.00004946) | 0 | 0.00025249 (0.00013073, 0.00037844) | 0 |
| Dehydroacetic Acid → Acetylcholine | 0.00026440 (0.00017096, 0.00037474) | 0 | 0.00003312 (0.00001365, 0.00005340) | 0.002 | 0.00023128 (0.00014296, 0.00033976) | 0 |
| Dehydroacetic Acid → Glutamic acid | 0.00022451 (0.00008210, 0.00038047) | 0.006 | 0.00005476 (0.00003392, 0.00007744) | 0 | 0.00016974 (0.00002439, 0.00032504) | 0.02 |
| Dehydroacetic Acid → Histidine | 0.00038865 (0.00027003, 0.00049839) | 0 | 0.00003190 (0.00001707, 0.00005001) | 0 | 0.00035675 (0.00024292, 0.00046899) | 0 |
| Dehydroacetic Acid → Serylleucine | 0.00037776 (0.00031360, 0.00042768) | 0 | 0.00001953 (0.00000547, 0.00003594) | 0.004 | 0.00035823 (0.00029335, 0.00041146) | 0 |
| Dehydroacetic Acid → Arginylleucine | 0.00031304 (0.00024084, 0.00037439) | 0 | 0.00001980 (0.00000599, 0.00003599) | 0.002 | 0.00029324 (0.00021926, 0.00036066) | 0 |
| Dehydroacetic Acid → N-Lactoylphenylalanine | 0.00029867 (0.00019287, 0.00039742) | 0 | 0.00004455 (0.00002337, 0.00006693) | 0 | 0.00025412 (0.00015409, 0.00035427) | 0 |
| Dehydroacetic Acid → Kynurenine | 0.00029300 (0.00009475, 0.00049872) | 0.002 | 0.00002663 (0.00000918, 0.00004275) | 0.006 | 0.00026638 (0.00007220, 0.00047359) | 0.006 |
| Dehydroacetic Acid → Spermine | 0.00020449 (0.00009257, 0.00032015) | 0 | 0.00004083 (0.00002150, 0.00006362) | 0 | 0.00016366 (0.00004888, 0.00027514) | 0.004 |
| Dehydroacetic Acid → Cer(d18:2/18:1) | 0.00034870 (0.00028783, 0.00040183) | 0 | 0.00001518 (0.00000269, 0.00003010) | 0.022 | 0.00033353 (0.00026977, 0.00039227) | 0 |
| Dehydroacetic Acid → Cer(d18:2/20:0) | 0.00035061 (0.00029182, 0.00040403) | 0 | 0.00001506 (0.00000168, 0.00002886) | 0.026 | 0.00033555 (0.00027432, 0.00039061) | 0 |
| Dehydroacetic Acid → Cer(d20:1/PGD2) | 0.00021572 (0.00003418, 0.00040269) | 0.02 | 0.00005410 (0.00003213, 0.00007676) | 0 | 0.00016163 (-0.00002173, 0.00034134) | 0.07 |
| Dehydroacetic Acid → Dioctylamine | 0.00023529 (0.00012533, 0.00034116) | 0 | 0.00003471 (0.00001481, 0.00005411) | 0 | 0.00020059 (0.00008298, 0.00030605) | 0 |
| Dehydroacetic Acid → Homocystamine | 0.00011059 (0.00004310, 0.00018267) | 0 | 0.00006161 (0.00003636, 0.00008924) | 0 | 0.00004898 (-0.00001669, 0.00012233) | 0.14 |
| Dehydroacetic Acid → Hexanoylcarnitine | 0.00015997 (0.00004291, 0.00028213) | 0.01 | 0.00005364 (0.00002965, 0.00007886) | 0 | 0.00010633 (-0.00000584, 0.00022407) | 0.062 |
| Dehydroacetic Acid → 6-Octenoylcarnitine | 0.00036201 (0.00004003, 0.00066625) | 0.032 | 0.00004539 (0.00002475, 0.00006703) | 0 | 0.00031663 (0.00000224, 0.00063170) | 0.05 |
| Dehydroacetic Acid → Hexadecasphingosine | 0.00023178 (0.00011439, 0.00035732) | 0.002 | 0.00003699 (0.00001689, 0.00005827) | 0.002 | 0.00019479 (0.00007845, 0.00032218) | 0.004 |
| Dehydroacetic Acid → Isobutyryl-L-carnitine | 0.00029203 (0.00000343, 0.00057718) | 0.048 | 0.00003795 (0.00001911, 0.00005920) | 0 | 0.00025407 (-0.00003308, 0.00053256) | 0.084 |
| Dehydroacetic Acid → L-Acetylcarnitine | 0.00030164 (0.00017570, 0.00042918) | 0 | 0.00002449 (0.00000587, 0.00004373) | 0.006 | 0.00027715 (0.00015320, 0.00040551) | 0 |
| Dehydroacetic Acid → non-3-enedioic acid | 0.00038449 (0.00013738, 0.00061558) | 0.002 | 0.00002904 (0.00001181, 0.00004571) | 0.002 | 0.00035545 (0.00010857, 0.00058563) | 0.002 |
| Dehydroacetic Acid → Sphingosine | 0.00034512 (0.00022393, 0.00047654) | 0 | 0.00003292 (0.00001571, 0.00005121) | 0 | 0.00031220 (0.00019034, 0.00044665) | 0 |
| Dehydroacetic Acid → Sebacic acid | 0.00034801 (0.00018441, 0.00050918) | 0 | 0.00001846 (0.00000645, 0.00003431) | 0.01 | 0.00032956 (0.00017017, 0.00049286) | 0 |
| Dehydroacetic Acid → Palmitic amide | 0.00022917 (0.00013061, 0.00033901) | 0 | 0.00004794 (0.00002803, 0.00006906) | 0 | 0.00018122 (0.00008226, 0.00029238) | 0 |
| Dehydroacetic Acid → Palmitoleamide | 0.00020602 (0.00014395, 0.00027667) | 0 | 0.00003299 (0.00001071, 0.00005417) | 0.002 | 0.00017303 (0.00011658, 0.00024400) | 0 |
| Dehydroacetic Acid → 4-Oxo-L-proline | 0.00015114 (0.00009178, 0.00021749) | 0 | 0.00004358 (0.00002189, 0.00006648) | 0 | 0.00010756 (0.00005283, 0.00017049) | 0 |
| Dehydroacetic Acid → PA(i-21:0/8:0) | 0.00022226 (0.00005636, 0.00039300) | 0.012 | 0.00004425 (0.00001768, 0.00007069) | 0.002 | 0.00017801 (0.00001515, 0.00034791) | 0.026 |
| Dehydroacetic Acid → PA(PGE2/P-16:0) | 0.00037293 (0.00026546, 0.00048034) | 0 | 0.00002395 (0.00000866, 0.00004111) | 0.008 | 0.00034898 (0.00023181, 0.00045803) | 0 |
| Dehydroacetic Acid → PA(TXB2/i-17:0) | 0.00020920 (0.00009886, 0.00032845) | 0 | 0.00005107 (0.00003124, 0.00007294) | 0 | 0.00015813 (0.00005063, 0.00028075) | 0.006 |
| Dehydroacetic Acid → PA(LTE4/13:0) | 0.00021854 (0.00012049, 0.00030949) | 0.002 | 0.00005356 (0.00003186, 0.00007752) | 0 | 0.00016498 (0.00006345, 0.00025763) | 0.004 |
| Dehydroacetic Acid → LysoPC(28:0/0:0) | 0.00019815 (0.00007177, 0.00034496) | 0.006 | 0.00005002 (0.00002969, 0.00007254) | 0 | 0.00014813 (0.00001389, 0.00029351) | 0.034 |
| Dehydroacetic Acid → LysoPC(17:0/0:0) | 0.00031458 (0.00018418, 0.00045236) | 0 | 0.00001762 (0.00000322, 0.00003271) | 0.018 | 0.00029696 (0.00016621, 0.00044318) | 0 |
| Dehydroacetic Acid → PC(18:1(9Z)e/2:0) | 0.00032770 (0.00018941, 0.00045482) | 0 | 0.00004424 (0.00002477, 0.00006636) | 0 | 0.00028346 (0.00013981, 0.00041500) | 0 |
| Dehydroacetic Acid → LysoPE(P-18:0/0:0) | 0.00036200 (0.00029656, 0.00041060) | 0 | 0.00001277 (0.00000106, 0.00002534) | 0.032 | 0.00034923 (0.00028310, 0.00040153) | 0 |
| Dehydroacetic Acid → PE(18:2(9Z,12Z)/14:0) | 0.00022134 (0.00000269, 0.00046084) | 0.046 | 0.00004606 (0.00002358, 0.00007027) | 0 | 0.00017528 (-0.00004289, 0.00041591) | 0.144 |

**Supplementary Table 8.** The comparison of the counts of inflammatory cells in the sampled lung tissue areas (*P* values).

|  | **Ast** | **Ace** | **Sac** | **Ben** | **Mix** |
| --- | --- | --- | --- | --- | --- |
| **Con** | 0.0017 | <0.001 | <0.001 | <0.001 | 0.0515 |
| **Tol** | 0.0309 | 0.0008 | 0.0034 | <0.001 | 0.1835 |

Note: Con: the controls. Ast: the asthma model. Tol: the oral tolerance in asthma model. Ace: acesulfame-treated group. Sac: sodium saccharin-treated group. Ben: sodium benzoate-treated group. Mix: mixture-treated group (Ace + Sac + Ben). n=4.

**Supplementary Table 9.** The comparison of the proportion of helper T cells and DCs in the tissue of murine lung (*P* values).

| **Th1** |  | **Ast** | **Ace** | **Sac** | **Ben** | **Mix** |
| --- | --- | --- | --- | --- | --- | --- |
|  | **Con** | 0.0586 | 0.1078 | 0.0894 | 0.0526 | 0.0664 |
|  | **Tol** | 0.0497 | 0.5004 | 0.3124 | 0.0341 | 0.1078 |
| **Th2** |  | **Ast** | **Ace** | **Sac** | **Ben** | **Mix** |
|  | **Con** | 0.0250 | 0.0143 | 0.0144 | 0.0153 | 0.0201 |
|  | **Tol** | 0.0351 | 0.0236 | 0.0227 | 0.0337 | 0.0228 |
| **Th17** |  | **Ast** | **Ace** | **Sac** | **Ben** | **Mix** |
|  | **Con** | 0.0787 | 0.0765 | 0.0442 | 0.095 | 0.1049 |
|  | **Tol** | 0.0369 | 0.0074 | 0.0209 | 0.0306 | 0.0583 |
| **Treg** |  | **Ast** | **Ace** | **Sac** | **Ben** | **Mix** |
|  | **Con** | 0.0131 | 0.0133 | 0.0202 | 0.0182 | 0.0169 |
|  | **Tol** | 0.104 | 0.0772 | 0.1912 | 0.1532 | 0.1823 |
| **Allergic DC** |  | **Ast** | **Ace** | **Sac** | **Ben** | **Mix** |
|  | **Con** | 0.0002 | 0.0032 | 0.0059 | 0.0036 | 0.0003 |
|  | **Tol** | 0.0009 | 0.0054 | 0.0079 | 0.0063 | 0.0008 |
| **Tolerogenic DC** |  | **Ast** | **Ace** | **Sac** | **Ben** | **Mix** |
|  | **Con** | 0.0270 | 0.5047 | 0.0538 | 0.2521 | 0.0244 |
|  | **Tol** | 0.0674 | 0.1839 | 0.0772 | 0.0974 | 0.0765 |
| **Th1/Th2** |  | **Ast** | **Ace** | **Sac** | **Ben** | **Mix** |
|  | **Con** | 0.084 | 0.0947 | 0.092 | 0.0882 | 0.0768 |
|  | **Tol** | 0.0176 | 0.0383 | 0.0275 | 0.0274 | 0.0166 |
| **Th17/Treg** |  | **Ast** | **Ace** | **Sac** | **Ben** | **Mix** |
|  | **Con** | 0.0031 | 0.0014 | 0.0041 | 0.0104 | 0.0299 |
|  | **Tol** | 0.0014 | 0.0003 | 0.0018 | 0.0079 | 0.0319 |
| **Allergic DC/Tolerogenic DC** |  | **Ast** | **Ace** | **Sac** | **Ben** | **Mix** |
|  | **Con** | 0.0023 | 0.0047 | 0.0214 | 0.0160 | 0.0004 |
|  | **Tol** | 0.0019 | 0.0044 | 0.0224 | 0.0167 | 0.0003 |

Note: Con: the controls. Ast: the asthma model. Tol: the oral tolerance in asthma model. Ace: acesulfame-treated group. Sac: sodium saccharin-treated group. Ben: sodium benzoate-treated group. Mix: mixture-treated group (Ace + Sac + Ben). n=5.

**Supplementary Table 10.** The comparison of the proportion of helper T cells and DCs in the tissue of murine MLN (*P* values).

| **Th1** |  | **Ast** | **Ace** | **Sac** | **Ben** | **Mix** |
| --- | --- | --- | --- | --- | --- | --- |
|  | **Con** | 0.0799 | 0.0598 | 0.0420 | 0.0264 | 0.0249 |
|  | **Tol** | 0.0325 | 0.0293 | 0.0300 | 0.0255 | 0.0250 |
| **Th2** |  | **Ast** | **Ace** | **Sac** | **Ben** | **Mix** |
|  | **Con** | 0.092 | 0.0287 | 0.1174 | 0.0475 | 0.0275 |
|  | **Tol** | 0.0801 | 0.0227 | 0.0843 | 0.0368 | 0.0226 |
| **Th17** |  | **Ast** | **Ace** | **Sac** | **Ben** | **Mix** |
|  | **Con** | 0.1026 | 0.0654 | 0.0281 | 0.3175 | 0.1474 |
|  | **Tol** | 0.0075 | 0.0268 | 0.0115 | 0.0267 | 0.0635 |
| **Treg** |  | **Ast** | **Ace** | **Sac** | **Ben** | **Mix** |
|  | **Con** | 0.0044 | 0.0086 | 0.0485 | 0.5214 | 0.3888 |
|  | **Tol** | 0.0305 | 0.0360 | 0.0385 | 0.162 | 0.0687 |
| **Allergic DC** |  | **Ast** | **Ace** | **Sac** | **Ben** | **Mix** |
|  | **Con** | 0.0050 | 0.0585 | 0.0864 | 0.1012 | 0.0762 |
|  | **Tol** | 0.131 | 0.1573 | 0.9165 | 0.2522 | 0.2169 |
| **Tolerogenic DC** |  | **Ast** | **Ace** | **Sac** | **Ben** | **Mix** |
|  | **Con** | 0.0708 | 0.0633 | 0.0721 | 0.0921 | 0.1095 |
|  | **Tol** | 0.0083 | 0.0070 | 0.0073 | 0.0065 | 0.0072 |
| **Th1/Th2** |  | **Ast** | **Ace** | **Sac** | **Ben** | **Mix** |
|  | **Con** | 0.0976 | 0.0912 | 0.1117 | 0.0848 | 0.0783 |
|  | **Tol** | 0.0388 | 0.0376 | 0.0416 | 0.0365 | 0.0351 |
| **Th17/Treg** |  | **Ast** | **Ace** | **Sac** | **Ben** | **Mix** |
|  | **Con** | 0.0155 | 0.0360 | 0.0175 | 0.4665 | 0.3303 |
|  | **Tol** | 0.0076 | 0.0226 | 0.0113 | 0.0330 | 0.2738 |
| **Allergic DC/Tolerogenic DC** |  | **Ast** | **Ace** | **Sac** | **Ben** | **Mix** |
|  | **Con** | 0.0097 | 0.0326 | 0.0173 | 0.2877 | 0.0321 |
|  | **Tol** | 0.0096 | 0.0340 | 0.0179 | 0.2946 | 0.0346 |

Note: Con: the controls. Ast: the asthma model. Tol: the oral tolerance in asthma model. Ace: acesulfame-treated group. Sac: sodium saccharin-treated group. Ben: sodium benzoate-treated group. Mix: mixture-treated group (Ace + Sac + Ben). n=5.

**Supplementary Table 11.** The differential metabolites in mouse MLN CD4^+^ cells among groups.

| **Groups** | **Increased metabolites (HMDB ID)** | **Decreased metabolites (HMDB ID)** |
| --- | --- | --- |
| Acesulfame *vs.* Tolerance | - MG(PGF1alpha/0:0/0:0) (HMDB0260511) - tridec-4-enoic acid (HMDB0340740) - PG(18:1(12Z)-2OH(9,10)/i-13:0) (HMDB0271208) - Platelet-activating factor (HMDB0062195) - PA(22:4(7Z,10Z,13Z,16Z)/15:0) (HMDB0115325) - N-Palmitoyl Leucine (HMDB0241928) - 2,3-Dipalmitoyl-S-glycerylcysteine (HMDB0256098) - Cyclohexaneundecanoic acid (HMDB0030997) - Tocophersolan (HMDB0034354) - PC(36:4) (HMDB0007889) - (13Z,16Z)-tetracosa-13,16-dienoic acid (HMDB0340949) - SM(d18:1/20:3(8Z,11Z,14Z)-2OH(5,6)) (HMDB0290525) - DG(20:3(6,8,11)-OH(5)/0:0/8:0) (HMDB0297262) - Docosadienoic acid (HMDB0251556) - PG(20:4(6E,8Z,11Z,14Z)+=O(5)/i-14:0) (HMDB0271304) - Na,Na-Dimethylhistamine (HMDB0033438) - LysoPI(20:4(5Z,8Z,11Z,14Z)/0:0) (HMDB0061690) - LysoPE(P-16:0/0:0) (HMDB0011152) | - 3,9-Dihydroxydodecanoic acid (HMDB0340723) - PS(14:0/5-iso PGF2VI) (HMDB0280843) - L-Tyrosine (HMDB0000158) - Propyl propionate (HMDB0030059) - Undeca-6,8-dienoylcarnitine (HMDB0241189) - Trideca-3,5,7-trienoylcarnitine (HMDB0241339) - SM(d18:1/16:1) (HMDB0240613) - N-Oleoyl GABA (HMDB0062335) - Dibutyl adipate (HMDB0251172) - Hydroxysphingomyeline C16:1 (HMDB0013463) - Cer(d18:2/20:0) (HMDB0341548) |
| Sodium saccharin *vs.* Tolerance | - MG(PGF1alpha/0:0/0:0) (HMDB0260511) - Spermidine (HMDB0001257) - L-Acetylcarnitine (HMDB0000201) - Phosphorylcholine (HMDB0001565) - 5'-Methylthioadenosine (HMDB0001173) - PG(18:1(12Z)-2OH(9,10)/i-13:0) (HMDB0271208) - Platelet-activating factor (HMDB0062195) - N-Palmitoyl Leucine (HMDB0241928) - PA(22:4(7Z,10Z,13Z,16Z)/15:0) (HMDB0115325) - PE-NMe2(18:1(9Z)/15:0) (HMDB0114040) - (5Z)-pentadec-5-enoic acid (HMDB0340860) - Cyclohexaneundecanoic acid (HMDB0030997) - Tocophersolan (HMDB0034354) - (13Z,16Z)-tetracosa-13,16-dienoic acid (HMDB0340949) - SM(d18:1/20:3(8Z,11Z,14Z)-2OH(5,6)) (HMDB0290525) - DG(20:3(6,8,11)-OH(5)/0:0/8:0) (HMDB0297262) - Docosadienoic acid (HMDB0251556) - Cholesteryl acetate (HMDB0003822) - DG(20:3(5Z,11Z,14Z)-O(8,9)/16:0/0:0) (HMDB0295400) - GlcCer(d18:1/22:0) (HMDB0004974) - N-Acetyltryptamine (HMDB0255077) - LysoPI(20:4(5Z,8Z,11Z,14Z)/0:0) (HMDB0061690) - LysoPE(P-16:0/0:0) (HMDB0011152) | - Dibutyl adipate (HMDB0251172) - CerP(d18:1/22:0) (HMDB0010703) - PS(14:0/5-iso PGF2VI) (HMDB0280843) - Pro-Ile (HMDB0304810) - L-Tyrosine (HMDB0000158) - Propyl propionate (HMDB0030059) - SM(d18:1/16:1) (HMDB0240613) - N-Oleoyl GABA (HMDB0062335) |
| Sodium benzoate *vs.* Tolerance | - MG(PGF1alpha/0:0/0:0) (HMDB0260511) - L-Tyrosine (HMDB0000158) - 3-Mercaptohexyl hexanoate (HMDB0037766) - 3-Methylheptanoylcarnitine (HMDB0241041) - PG(18:1(12Z)-2OH(9,10)/i-13:0) (HMDB0271208) - Platelet-activating factor (HMDB0062195) - N-Palmitoyl Leucine (HMDB0241928) - Dibutyl adipate (HMDB0251172) - 2,3-Dipalmitoyl-S-glycerylcysteine (HMDB0256098) - Cyclohexaneundecanoic acid (HMDB0030997) - PC(36:4) (HMDB0007889) - (13Z,16Z)-tetracosa-13,16-dienoic acid (HMDB0340949) - SM(d18:1/20:3(8Z,11Z,14Z)-2OH(5,6)) (HMDB0290525) - Docosadienoic acid (HMDB0251556) - 2,3-Diacetoxypropyl stearate (HMDB0059931) - Cholesteryl acetate (HMDB0003822) - LysoPI(20:4(5Z,8Z,11Z,14Z)/0:0) (HMDB0061690) | - DG(PGD1/0:0/i-18:0) (HMDB0299746) - CerP(d18:1/22:0) (HMDB0010703) - PS(14:0/5-iso PGF2VI) (HMDB0280843) - L-Tyrosine (HMDB0000158) - 2-Phenylethyl pentanoate (HMDB0035016) - Propyl propionate (HMDB0030059) - Undeca-6,8-dienoylcarnitine (HMDB0241189) - Trideca-3,5,7-trienoylcarnitine (HMDB0241339) - SM(d18:1/16:1) (HMDB0240613) - SM(d18:1/16:1) (HMDB0240613) - N-Oleoyl GABA (HMDB0062335) - Hydroxysphingomyeline C16:1 (HMDB0013463) - Cer(d18:2/20:0) (HMDB0341548) - PE(14:1(9Z)/15:0) (HMDB0008856) - DG(22:1n9/0:0/20:4n6) (HMDB0056257) |
| Mix *vs.* Tolerance | - MG(PGF1alpha/0:0/0:0) (HMDB0260511) - L-Tyrosine (HMDB0000158) - 3-Methylheptanoylcarnitine (HMDB0241041) - PG(18:1(12Z)-2OH(9,10)/i-13:0) (HMDB0271208) - N-Palmitoyl Leucine (HMDB0241928) - 2,3-Dipalmitoyl-S-glycerylcysteine (HMDB0256098) - Cyclohexaneundecanoic acid (HMDB0030997) - Tocophersolan (HMDB0034354) - PC(36:4) (HMDB0007889) - (13Z,16Z)-tetracosa-13,16-dienoic acid (HMDB0340949) - SM(d18:1/20:3(8Z,11Z,14Z)-2OH(5,6)) (HMDB0290525) - PG(20:4(6E,8Z,11Z,14Z)+=O(5)/i-14:0) (HMDB0271304) | - CerP(d18:1/22:0) (HMDB0010703) - PS(14:0/5-iso PGF2VI) (HMDB0280843) - 2-Phenylethyl pentanoate (HMDB0035016) - Propyl propionate (HMDB0030059) - Undeca-6,8-dienoylcarnitine (HMDB0241189) - Trideca-3,5,7-trienoylcarnitine (HMDB0241339) - Dibutyl adipate (HMDB0251172) - Hydroxysphingomyeline C16:1 (HMDB0013463) - PE(14:1(9Z)/15:0) (HMDB0008856) - DG(22:1n9/0:0/20:4n6) (HMDB0056257) - DG(PGD1/0:0/i-18:0) (HMDB0299746) |

- 1. **Supplementary Figures**


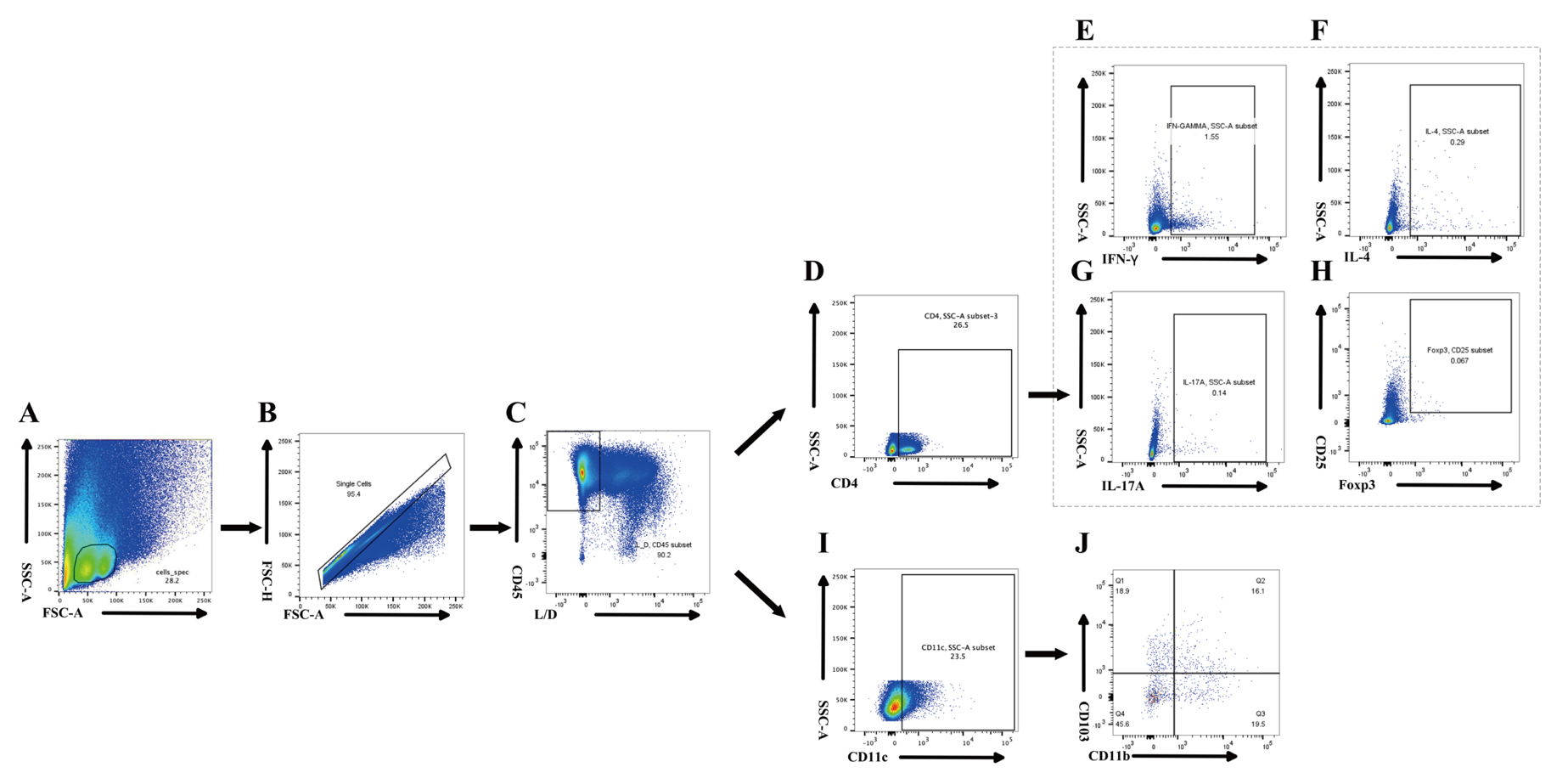


**Supplementary Figure 1.** Gating strategy for CD4^+^ T cells and DCs. Immune cells were gated accordingly to forward and side scatter (A)Doublets (B) and dead cells (C) were first excluded from the analysis. Immune cells were defined as CD45^+^ (C), and CD4^+^ T cells were defined as CD4^+^. Th1, Th2, Th17 and Treg cells were identified by the expression of IFN-γ (E), IL-4 (F), IL-17A (G), and CD25 and FOXP3 (H), respectively. DCs were defined as CD11c^+^ (I). CD103 and CD11b expression for cells pre-gated on Tolerance DC (J-Q1) and Allergy DC (J-Q2 and J-Q3).
